# Bipartite interface of the measles virus phosphoprotein X domain with the large polymerase protein regulates viral polymerase dynamics

**DOI:** 10.1371/journal.ppat.1007995

**Published:** 2019-08-05

**Authors:** Venice Du Pont, Yi Jiang, Richard K. Plemper

**Affiliations:** 1 Institute for Biomedical Sciences, Georgia State University, Atlanta, Georgia, United States of America; 2 Department of Mathematics and Statistics, Georgia State University, Atlanta, Georgia, United States of America; Harvard Medical School, UNITED STATES

## Abstract

Measles virus (MeV) is a highly contagious, re-emerging, major human pathogen. Replication requires a viral RNA-dependent RNA polymerase (RdRP) consisting of the large (L) polymerase protein complexed with the homo-tetrameric phosphoprotein (P). In addition, P mediates interaction with the nucleoprotein (N)-encapsidated viral RNA genome. The nature of the P:L interface and RdRP negotiation of the ribonucleoprotein template are poorly understood. Based on biochemical interface mapping, swapping of the central P tetramerization domain (OD) for yeast GCN4, and functional assays, we demonstrate that the MeV P-to-L interface is bipartite, comprising a coiled-coil microdomain proximal to the OD and an unoccupied face of the triangular prism-shaped C-terminal P X-domain (P-XD), which is distinct from the known P-XD face that binds N-tail. Mixed null-mutant P tetramers regained L-binding competence in a ratio-dependent manner and fully reclaimed bioactivity in minireplicon assays and recombinant MeV, demonstrating that the individual L-binding interface elements are physically and mechanistically distinct. P-XD binding competence to L and N was likewise trans-complementable, which, combined with mathematical modeling, enabled the mechanistic characterization of P through two- and stoichiometrically-controlled three-way complementations. Only one each of the four XDs per P tetramer must be L or N binding-competent for bioactivity, but interaction of the same P-XD with L and N was mutually exclusive, and L binding superseded engaging N. Mixed P tetramers with a single, designated L binding-competent P-XD caused significant RdRP hyperactivity, outlining a model of iterative resolution and reformation of the P-XD:L interface regulating polymerase mobility.

## Author summary

MeV belongs to the order of non-segmented negative polarity RNA viruses, which includes devastating human pathogens. While all feature encapsidated RNA genomes and P-L type polymerase complexes, insight into the intermolecular interactions within the polymerase hetero-oligomer and between the polymerase complex and the RNA-encapsidating N protein is rudimentary. Our mapping of the MeV P-to-L interaction revealed a bipartite interface with physically and mechanistically distinct contact zones, which provided a unique experimental platform to dissect the stoichiometry and dynamics of P interactions with L and N through functional trans-complementation assays in minireplicon settings and, ensuring physiological significance, recombinant virions. The identification and functional characterization of a novel L-binding face on the P-XD triangular prism, distinct from the side contacting N-tail, places P-XD at the center of a regulatory mechanism that controls the dynamics of polymerase advancement along the encapsidated genome through iterative separation and restoration of P-XD interaction with L. These observations and the high structural homology of polymerase components within the *Paramyxoviridae* recommend the P-XD:L protein-protein interface as premier target for directed drug discovery against emerging and re-emerging paramyxoviruses.

## Introduction

Non-segmented negative polarity RNA viruses comprise the etiological agents of some of the most devastating acute viral infections, such as Ebola virus, rabies virus, and, of the paramyxovirus family, Nipah virus and MeV. Common to all viruses in this order, the single-stranded RNA genomes are wrapped by nucleocapsid (N) proteins into helical ribonucleoprotein (RNP) complexes, and encapsidated RNAs are exclusively recognized as templates for transcription and replication by the viral RNA-dependent RNA-polymerases (RdRPs) complexes [1].

The core components of these RdRPs are the viral large (L) and phospho- (P) proteins [2–4]. L contains all catalytic centers required for polymerization and viral mRNA capping and methylation, while P functions as an essential L chaperone, tethering L to the RNP through direct interactions with both L and N proteins [5–9]. In addition, P forms N^0^-P complexes with newly synthesized free N, preventing premature N multimerization and ensuring delivery of N to the replication site for co-transcriptional encapsidation of nascent genomic and antigenomic viral RNAs [10–18]. Although its interaction with both L and N predestines P for a major regulatory role in RdRP activity [9], little is known about the dynamics of the interface between P and L in polymerase complexes and how a dynamic interaction may control polymerase function.

**Fig 1:**
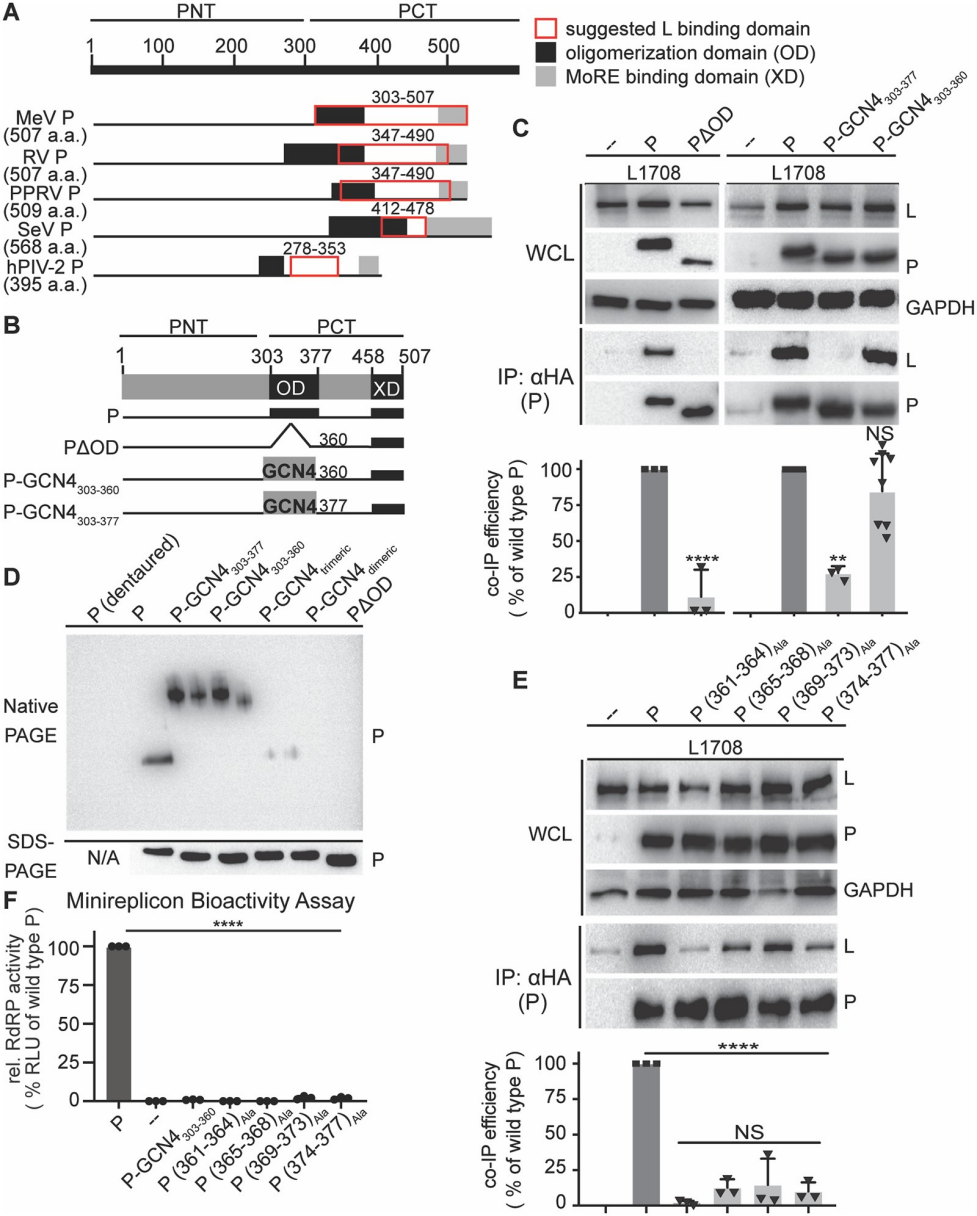
P OD C-terminal microdomain is required for P-to-L binding. **A)** Overview of proposed L binding domains on P for different *Paramyxoviridae*. Numbering refers to MeV P. **B)** Schematic representation of P with OD deletion or exchange with yeast GCN4. **C)** Immunoblots of whole cell lysates (WCL) and immunoprecipitates (IP) after co-transfection with L_1708_ (L) and P variants shown in (B). P was detected with anti-HA antibodies, L with anti-FLAG antibodies. GAPDH served as loading control. Graph shows relative co-IP efficiency of L with P; columns are means ± SD, symbols show individual biological repeats (n >= 3). **D)** Native PAGE analysis of P constructs shown in (B). Trimeric and dimeric variants of yeast GCN4 (P-GCN4_Trimeric_ and P-GCN4_Dimeric_, respectively) were included for mobility reference. SDS-PAGE shows immunoblots of the identical samples after denaturation and reduction. **E)** P-L co-IP of alanine scanning mutagenesis of the P (361–377) microdomain. **F)** Monocistronic minireplicon assay performed in the presence of wild type P or P mutants specified. Columns are means ± SD, symbols show individual biological repeats (n = 3). All statistical analyses through one-way ANOVA with Tukey’s post hoc multiple comparison test. (NS, not significant; **, p ≤ 0.01; ****: p ≤ 0.0001).

We have addressed this knowledge gap in the present study by example of the polymerase complexes of an archetype of the paramyxovirus family, MeV. As is characteristic for all paramyxovirus P proteins, MeV P has a modular organization in which three defined functional modules, an N-terminal domain, the central OD (residues 303–377), and the C-terminal P-XD (residues 459–507) are separated by large stretches of intrinsically disordered residues [19, 20] (Fig 1A). The N-terminal domain does not engage L but its first 50 residues bind to the globular core of the N protein, resulting in the formation of the N^0^-P complexes [21]. The OD mediates P oligomerization through assembly into parallel, α-helical coiled-coils, and minigenome reporter-based functional assays and structural studies have confirmed that the bioactive P oligomer is a homo-tetramer [22–24]. In the case of MeV P, a defined 3–4 heptad repeat motif spans the C-terminal region of OD uninterruptedly to residue 377.

P-XD does not tetramerize but stabilizes RdRP interaction with the RNP template through interaction with a molecular recognition element (MoRE) that is located in the largely unstructured C-terminal tail domain of the N protein [25, 26]. Co-crystal structures of MeV P-XD complexed with MoRE have revealed a stable 4-helix arrangement consisting of a single MoRE-derived α-helix and a triangular P-XD prism formed by three α-helices in a helix-turn-helix organization [27] (Fig 1A). The initial loading of the RdRP complex onto the RNP does not require MoRE binding [28], but subsequent interaction of P-XD with MoRE has been shown to prevent premature separation of the advancing polymerase from the template. A cartwheeling model was proposed that predicts iterative cycles of P-XD to MoRE binding and release to enable polymerase advancement along the template, while at all times maintaining at least one P-XD contact with the RNP [29, 30].

Large stretches of the C-terminal half of MeV P (PCT) including the OD have been implicated in contributing to P interaction with L [9, 31–33]. Slightly shorter sections were considered for the very closely related Rinderpest virus (RV) and peste des petits ruminants virus (PPRV), but the hypothetical P:L interfaces discussed still encompassed over 140 amino acids each [31, 34]. A stretch of charged amino acids in the central region of more distantly related Sendai virus (SeV) P OD was proposed to contact SeV L directly [35]. High structural plasticity in this area was attributed to a requirement for flexibility in the P-to-L interface during RNA synthesis [36]. In addition to SeV P OD, central kinks have been located in the OD sections of Nipah virus, Mumps virus, and MeV [23, 37, 38], and a positive contribution of this MeV P OD section to L binding was recently reported [9]. Unlike the P tetramerization domain-dependent formation of paramyxovirus polymerase complexes, short monomeric polypeptides derived from the N-terminal region of rhabdovirus P, spanning residues 41–61 in the case of vesicular stomatitis virus P [39] and 1–19 in the case of rabies virus P [40], are reportedly sufficient for biochemical detection of L binding. However, full L binding and stimulation of RdRP activity on nonencapsidated RNA templates has been shown to map to residues 41–106 of vesicular stomatitis virus P and 1–50 of rabies virus P [39, 41], indicating that the mononegavirus P:L interface is complex.

In this study, we have combined biochemical interface mapping with mechanistic assays to better understand the dynamic nature of the MeV P interface with L, probe a role of this interface in ensuring polymerase mobility along the RNP template, and explore determinants mediating spatiotemporal control of the process. Having identified a bipartite P to L contact zone consisting of a microdomain immediately downstream of the OD and an uncharacterized side of the P-XD prism that was previously considered unoccupied, we have examined the individual contributions of these microdomains to physical P interaction with L, interaction dynamics, and RdRP bioactivity in a groundbreaking trans-complementation assay of the distinct contact zones. Supported by mathematical models of experimentally observed trans-complementation profiles, our results reveal a multiplexed and highly dynamic interaction between P, L, and the RNA encapsidating N moieties that attributes a central regulatory role in orchestrating polymerase negotiation of the encapsidated template to P-XD. These data provide a framework for a revised mechanistic model of paramyxovirus RNA synthesis.

## Results

To dissect the contribution of individual MeV PCT domains to L binding, we first focused on OD. In MeV P expression plasmids, we either deleted this domain by removing residues 303–360 (OD structure up to residue 360 was resolved in [42]) or replaced this section up to residue 360 or 377 (end of defined heptad repeat motif), respectively, with the tetrameric mutant of the yeast general control protein 4 (GCN4) coiled-coil [43] (Fig 1B). The effect of these modifications on physical interaction of P with L was assessed through co-immunoprecipitation (co-IP) of the proteins from lysates of transiently transfected cells. All co-IPs were carried out with a C-terminally truncated form of L, L_1708_ lacking the L methyltransferase domain, that we have previously shown to be fully folding competent and capable of forming bioactive polymerase complexes when post-translationally combined with the corresponding C-terminal L fragment [44]. Co-IP efficiencies of L_1708_ or full-length L with P are indistinguishable (S1 Fig), but L_1708_ facilitates biochemical analysis of P-to-L binding due to a lower tendency to self-aggregate spontaneously [45].

### MeV P residues proximal to the structurally defined OD are critical for L interaction

When subjected to this assay, the PΔOD mutant lacking the oligomerization domain was unable to co-precipitate L (Fig 1C). In contrast, efficient wild type P-equivalent L binding was maintained when P OD up to residue 360 was replaced with yeast GCN4 (P-GCN4_303-360_). Further extension of the GCN4-substituted area up to P residue 377 eliminated any appreciable interaction with L. Native-PAGE analysis confirmed that both GCN4-substituted P mutants tetramerized efficiently, whereas PΔOD predictably remained monomeric (Fig 1D). In contrast to previous theories [23, 32, 37], residues in the MeV P OD core do not, therefore, form a physical interface with L but contribute to the interaction only indirectly through initiation of mandatory P tetramerization. However, residues in the short stretch at the OD C-terminus from position 361 to 377 are candidates for direct L binding.

Alanine-scanning mutagenesis of the 361–377 microdomain revealed that all sub-sections tested are required for efficient L binding (Fig 1E). Although reductions in L co-IP efficiency were statistically equivalent, we noted the lowest relative interaction with L when P residues 361–364 were substituted with alanines, creating P (361–364)_Ala_. Employing a mono-cistronic firefly luciferase MeV minireplicon reporter system that we have previously described [46], we assessed bioactivity of the different alanine mutants after co-expression with unmodified, homotypic MeV N and L proteins. Consistent with impaired physical interaction of these P mutants with L, all four constructs abolished RdRP bioactivity (Fig 1F). Despite its unaltered physical interaction with L, P-GCN4_303-360_ also lacked bioactivity in minireplicon assays, indicating that the P OD has a role in viral RdRP activity distinct from L binding and mediating P tetramerization.

### Substitutions in the P OD C-terminal microdomain are dominant-negative to RdRP bioactivity

**Fig 2:**
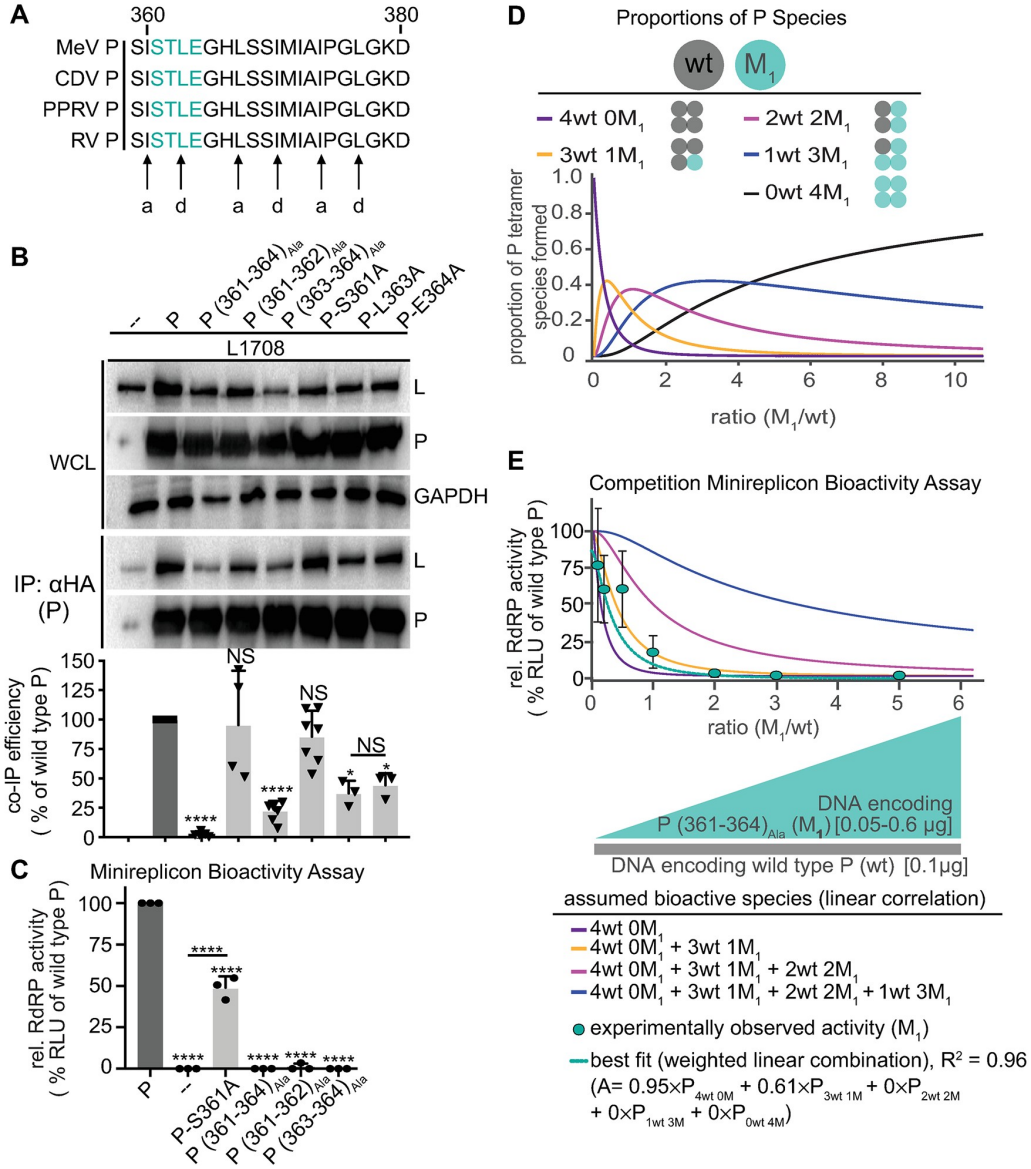
P (361–364)_Ala_ acts on RdRP bioactivity in a dominant-negative manner. **A)** Alignment of P residues 361–377 of selected *morbilliviruses*. Predicted a and d positions of heptad repeat motif are highlighted. **B)** P:L interaction analysis of P mutants with alanine substitutions at positions 361–364. Detection and quantitative analysis as in Fig 1C (n ≥ 3). **C)** Minireplicon assay with P alanine substitutions at positions 361–364 (n = 3). **D)** Schematic of mixed P tetramer species present after co-expression of P (361–364)_Ala_ (M_1_) and wild type P (wt). Equations specify the probability of formation for each tetramer species, and the graph shows the relative proportion of these species in co-transfected cells as a function of wild type and mutant input plasmid ratio, graphically represented below the x-axis. **E)** Observed RdRP activity in minireplicon assays in the presence of different P (361–364)_Ala_ and wt P ratios as depicted in (D). Symbols show means of experimentally observed biological repeats ± SD (n = 3). Solid lines represent activity curve predictions according to a linear combination model and the relative distributions of the P tetramer species shown in (D). The dotted line represents the best fit curve of the experimental data with weight assignments specified in the equation (A, relative RdRP activity), goodness of fit (R^2^) is indicated. All statistical analyses and symbols as detailed in Fig 1.

Residues in the P OD section 361–377 follow a 3–4 heptad repeat pattern that is characteristic for α-helical coiled-coils and conserved across pathogens of the morbillivirus genus (Fig 2A). To increase resolution of the alanine scan, we followed up with pairwise and individual substitutions in the 361–364 microdomain that had shown the strongest disruption of co-IP efficiency. This biochemical assessment demonstrated that disruption of P interaction with L was equally carried by residues 363 and 364, although the quadruple substitution still returned the most robust effect on average (Fig 2B). Independent of residual L binding capacity, all single and tandem substitutions except S361A eliminated RdRP bioactivity in minireplicon assays (Fig 2C).

Structural analysis has revealed that some helices of the P-OD coiled-coil extend to residue 373 [23]. Combined with an intact heptad repeat pattern up to residue 377, assembly of the entire 361–377 stretch into an extended coiled-coil and interaction with L as a tetrameric assembly appears likely. To test whether the P (361–364)_Ala_ substitution has a cooperative effect on P tetramer activity, we generated competition profiles of mixed P tetramers by gradually increasing the relative amount of mutant P in minireplicon assays in the presence of a constant, low level of wild type P. Under these conditions, five distinct P tetramer species will form in all co-transfected cells, with the relative species distribution determined by the input ratio of mutant and wild type P-encoding plasmid DNA (Fig 2D), since native-PAGE analysis confirmed that the 361–364 alanine substitution did not affect the ability of P to tetramerize (S2 Fig). Minireplicon-based assessment of RdRP bioactivity in the different cell populations revealed a steep decline in minireplicon expression in the presence of increasing amounts of the P (361–364)_Ala_ mutant (Fig 2E). This decline was not simply due to increasingly unfavorable ratios of P- versus N- and L-encoding plasmid transfected. When we generated P plasmid concentration-activity profiles, relative activities followed an optimum curve, but activity differences were statistically not-significant over the plasmid ratio range explored in the minigenome competition profile (S3 Fig).

We tested a series of mathematical models for the best description of the experimental competition data. Assigning all mutant and wild type P proteins co-expressed in the same cell equal probability to tetramerize, we considered mutant occupation of one P monomer to be independent of the other three monomers. Goodness of fit was excellent (R^2^ = 0.96) for a linear combination model in which only tetramers consisting of four wild type (4×wt) or three wild type and one mutant (3×wt/1×P (361–364)_Ala_) P monomers contribute to RdRP bioactivity, while all other P tetramer species are biologically inactive (Fig 2E). These results indicate that at most one P (361–364)_Ala_ monomer can be present in a partially bioactive P tetramer, revealed a dominant-negative effect of the P (361–364)_Ala_ mutation that we consider to be due to interference with coiled-coil extension.

### P-XD is an essential contributor to efficient P interaction with L

**Fig 3:**
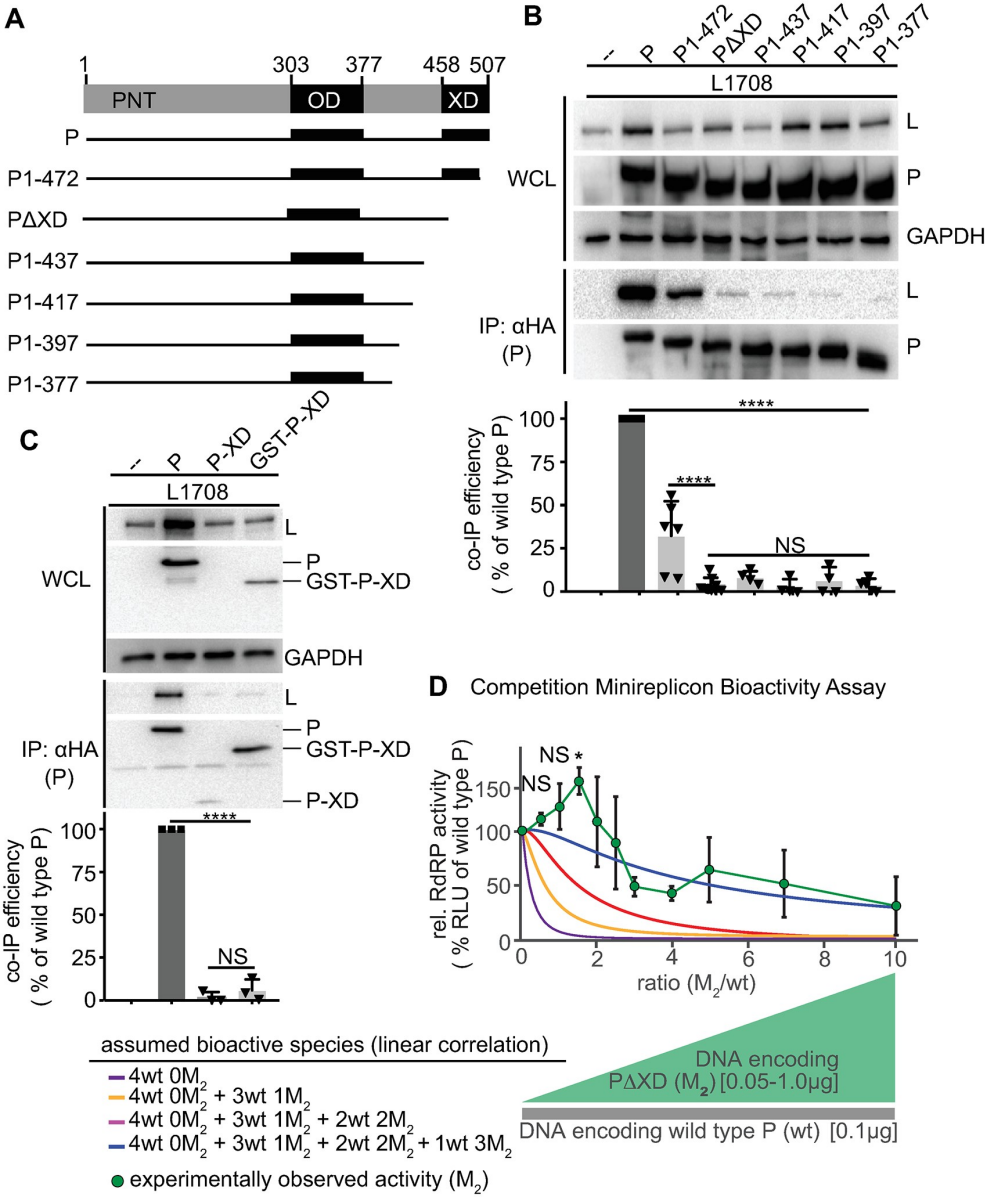
P-XD is essential for efficient P:L interaction, but P-XD deletion mutants lack cooperative negative impact on RdRP activity. **A**) Schematic of P mutants generated with C-terminal truncations. **B, C)** P:L interaction analysis of P mutants shown in (A) and P-XD expressed in isolation (P-XD) or as a GST fusion protein (GST-P-XD). Detection and quantitative analysis as in Fig 1C (n ≥4 (B) and n = 3 (C)). **D)** Observed RdRP activity in minireplicon assays in the presence of different PΔXD (M_2_) and wt P ratios as graphically depicted below the graph. Symbols with connecting line represent means of experimentally observed biological repeats ± SD (n = 3). Solid lines represent activity curve predictions according to a linear combination model as in Fig 2E. All statistical analyses and symbols as detailed in Fig 1 (*, p ≤ 0.05).

The C-terminal 50 residues of P form the XD, which has been shown biochemically and crystallographically to mediate binding of the polymerase complex to the RNP through interaction with the MoREs located near the end of each N-tail [5, 6, 27]. To confirm that P-XD has a direct role in L binding, we generated a series of mutants with C-terminal truncations of gradually increasing length (Fig 3A). Co-IP analyses identified an essential function of P-XD in mediating efficient interaction with L (Fig 3B), since even partial shortening of P-XD was sufficient to cause significant reduction in co-IP efficiency and larger truncations abolished all appreciable interaction between P and L. Consistent with a dual role of P-XD in both MoRE and L binding, all C-terminal P truncation mutants lacked bioactivity in minireplicon assays (S4 Fig). When we co-expressed isolated P-XDs—either in the form of individual polypeptides or as fusion proteins with GST for stabilization—with L, however, no biochemical interaction could be detected (Fig 3C). We conclude that P-XD is an essential contributor to efficient P-to-L binding. Unlike the strong interaction of P-XD with MoRE affording efficient co-immunoprecipitation [47], affinity of isolated P-XD polypeptides for L is low, isolated P-XDs are folding compromised, or P-XD does not share a direct interface with L.

To explore the minimal stoichiometry of intact XDs per P tetramer that is required for bioactivity, we again co-expressed increasing relative amounts of the PΔXD mutant with wild type P in minireplicon competition assays. The resulting activity profile was notably distinct from that obtained with the P (361–364)_Ala_ substitution (Fig 3D). Even at the highest relative ratios of the PΔXD mutant, RdRP activity remained significantly higher than background, indicating that PΔXD has no dominant-negative effect, and lower relative ratios of the PΔXD unexpectedly resulted in a significant boost in RdRP activity compared to that observed in the presence of wild type P only.

Modeling attempts revealed that these data could not be described with a weighted linear combination function. Therefore, we explored non-linear models, but the complex biology of P-XD prevented a satisfying mathematical representation of the experimental results without overfitting the data set. While our results thus reveal that partially reducing the number of L and MoRE binding-competent XDs within a P tetramer boosts overall RdRP bioactivity, meaningful mathematical modeling of the phenotype requires a better-targeted approach than removal of P-XD entirely. We therefore explored mapping of the P-XD interface contacting L.

### Distinct faces of the P-XD triangular prism mediate binding to MoRE and L

**Fig 4:**
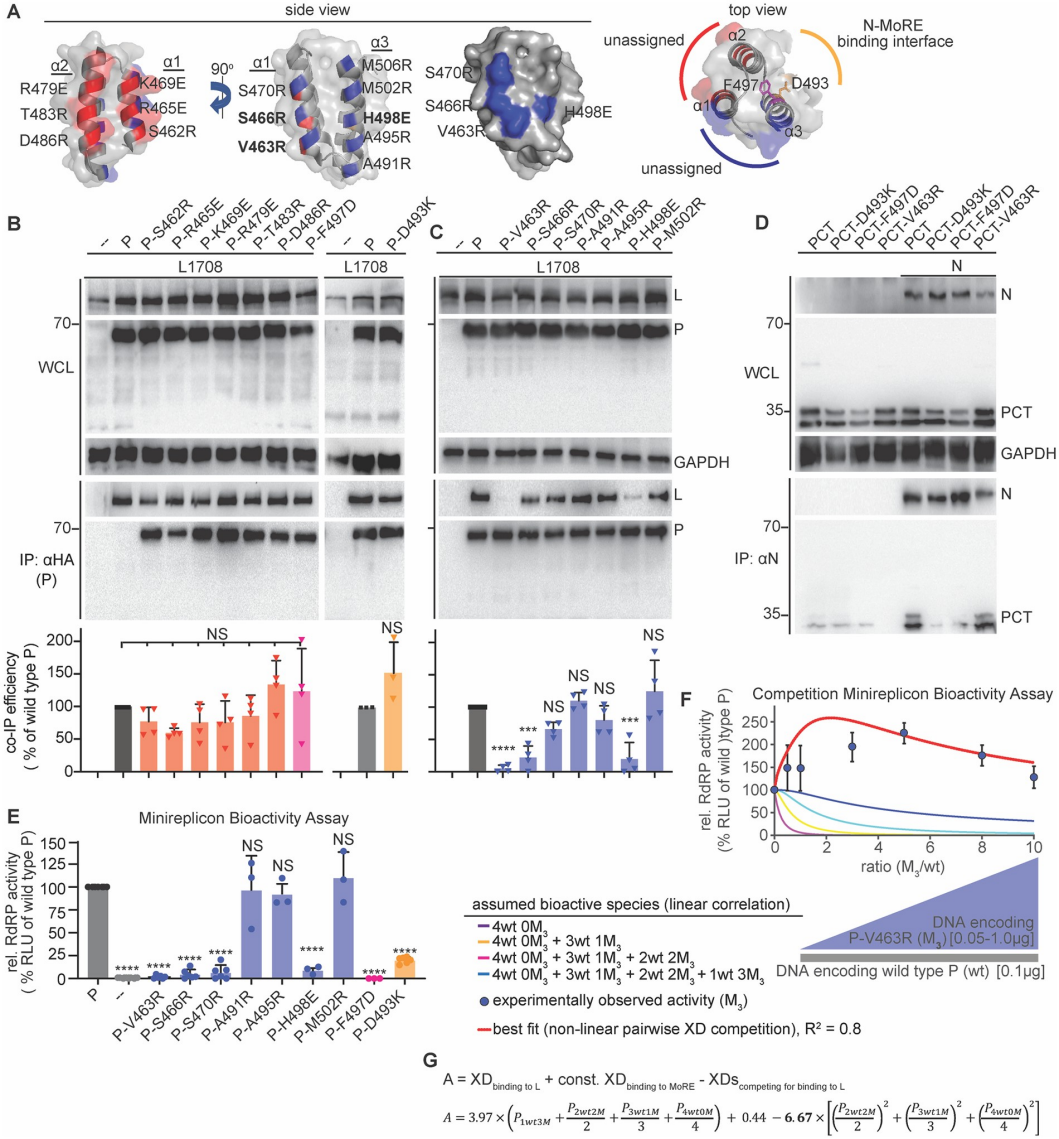
Identification of a specific L-binding face of the triangular prism fold of P-XD with regulatory effect on RdRP bioactivity. **A)** Structural representations of MeV P-XD (PDB: 1T6O; only the P-XD component is represented), shown in side and top views. Areas between helices α1/α2 (red) and α1/α3 (blue) form faces of the prism without known interaction partners or bioactivities. Specific residues on each face are specified. Top view shows the known MoRE-binding face of P-XD between helices α2/α3 (yellow), residues confirmed (F497) or predicted (D493) to respectively impair P-XD fold or MoRE binding when mutated are highlighted. Grey background represents space-fill surface model. **B, C)** P:L interaction analysis of P mutants with individual substitutions of residues specified in (A), forming the “red” and “blue” prism surfaces or implicated in MoRE binding (yellow). Detection and quantitative analysis as in Fig 1C (n = 4 (B); n = 3 (C)). **D)** PCT:N interaction after mutation of residue D493 on the MoRE interaction face of P-XD. Anti-N antibodies were used for IPs and anti-HA to detect PCT variants. **E)** Minireplicon analysis of RdRP activity in the presence of the specified P mutants. Columns represent means of experimentally observed values ± SD, symbols show individual biological repeats (n ≥ 3). **F)** Observed RdRP activity in minireplicon assays in the presence of different P-V463R (M_3_) and wt P ratios as graphically depicted below the graph. Symbols show means of experimentally observed biological repeats ± SD (n = 3). Solid lines represent activity curve predictions according to a linear combination model as in Fig 2E. The dotted line (red) represents the best fit curve of the experimental data based on a non-linear pairwise P-XD competition model, goodness of fit (R^2^) is indicated. **G)** Mathematical description of the model represented in (E). P-XD bioactivities considered and the corresponding weight assignments are specified. All statistical analyses and symbols as detailed in Fig 1 (***, p ≤ 0.001).

In co-crystals with MoRE, MeV P-XD assumed a helix-turn-helix fold of three α-helices approximately arranged as a triangular prism (Fig 4A). One side of this prism forms the interface with MoRE. To map individual residues mediating P-XD interaction with L, we targeted amino acids located at the surface of the other two sides of the prism through charge-reversal or charge-introducing substitutions, respectively. All of the resulting mutants were expressed efficiently (Figs 4B and 4C). Substitutions in the prism face between P-XD helices α1 and α2 did not affect L binding (Fig 4B), but three residues located in the face between α1 and α3 (V463 and S466 on α1, and H498 on α3) either abolished, or significantly reduced, P interaction with L (Fig 4C). Combined, these residues form a continuous microdomain covering the lower quadrant of the α1/α3 P-XD prism surface (Fig 4A). To test whether absence of the C-terminal methyltransferase domain in the L_1708_ fragment impacted co-IP results, we re-examined two P mutations abolishing interaction, P (361–364)_Ala_ and P-V463R, against full-length L. Neither P mutant precipitated L efficiently, validating the L_1708_-based results (S5 Fig).

We next asked whether the P-V463R substitution in α1 that caused the strongest reduction in co-IP efficiency specifically impairs P-to-L binding or globally alters the P-XD conformation. To address this question, we examined its effect on P-XD interaction with MoRE. Since N-terminal residues of P directly bind to Ncore [21], we generated and employed P C-terminal fragments (PCTs) starting upstream of the tetramerization domain at P residue 231 for this analysis. Co-IPs of wild type PCT and the PCT-V463R mutant with standard N revealed efficient binding, indicating that this mutation in the α1/α3 prism face does not affect interaction with MoRE and thus suggesting that global P-XD folding is intact (Fig 4D). The assay was validated through F497D or D493K substitutions, which are located in the hydrophobic core of P-XD between α2 and α3 [48] or predicted to reside on the surface of the MoRE-binding face of P-XD, respectively, and each indeed destroyed the interaction (Fig 4D).

Bioactivity testing of all P-XD mutants in minireplicon assays demonstrated a direct correlation between the effect of substitutions in the α1/α3 P-XD prism face on L binding and RdRP bioactivity (Fig 4E). α1 substitutions V463R and S466R and α3 mutation H498R in particular eliminated all polymerase activity, as did the F497D and D493K changes suppressing P-XD binding to MoRE. Competition profiles of the P-V463R mutant with wild type P revealed remarkable RdRP hyper-activity at higher relative amounts of the mutant, more than double that seen in the presence of wild type P alone (Fig 4F). Also this experimental data set was incompatible with a linear combination function.

We therefore considered again non-linear models, based on the following assumptions: all four P-XDs within a P tetramer function independently of each other; bioactivity requires XD binding to L and MoRE; both wild type and mutant XDs are equally MoRE binding competent; and the V463R mutation selectively impairs XD interaction with L. Most notably, the best fit mathematical description of the experimental data critically depends on the addition of a strong negative effect on bioactivity that arises from P-XD competition for L binding (Fig 4G). This model assumes that a single L binding-competent XD in the P tetramer is necessary and sufficient for bioactivity of the RdRP complex. Corroborating RdRP hyperactivity seen in the earlier PΔXD competition profiles, these results for the MoRE binding-competent but L binding-defective P-V463R mutant revealed that assignment of L-binding competence to only one XD per P tetramer creates conditions most favorable for overall RdRP activity.

### Trans-complementation of P mutants with distinct L binding deficiencies in minireplicon and recMeV

**Fig 5:**
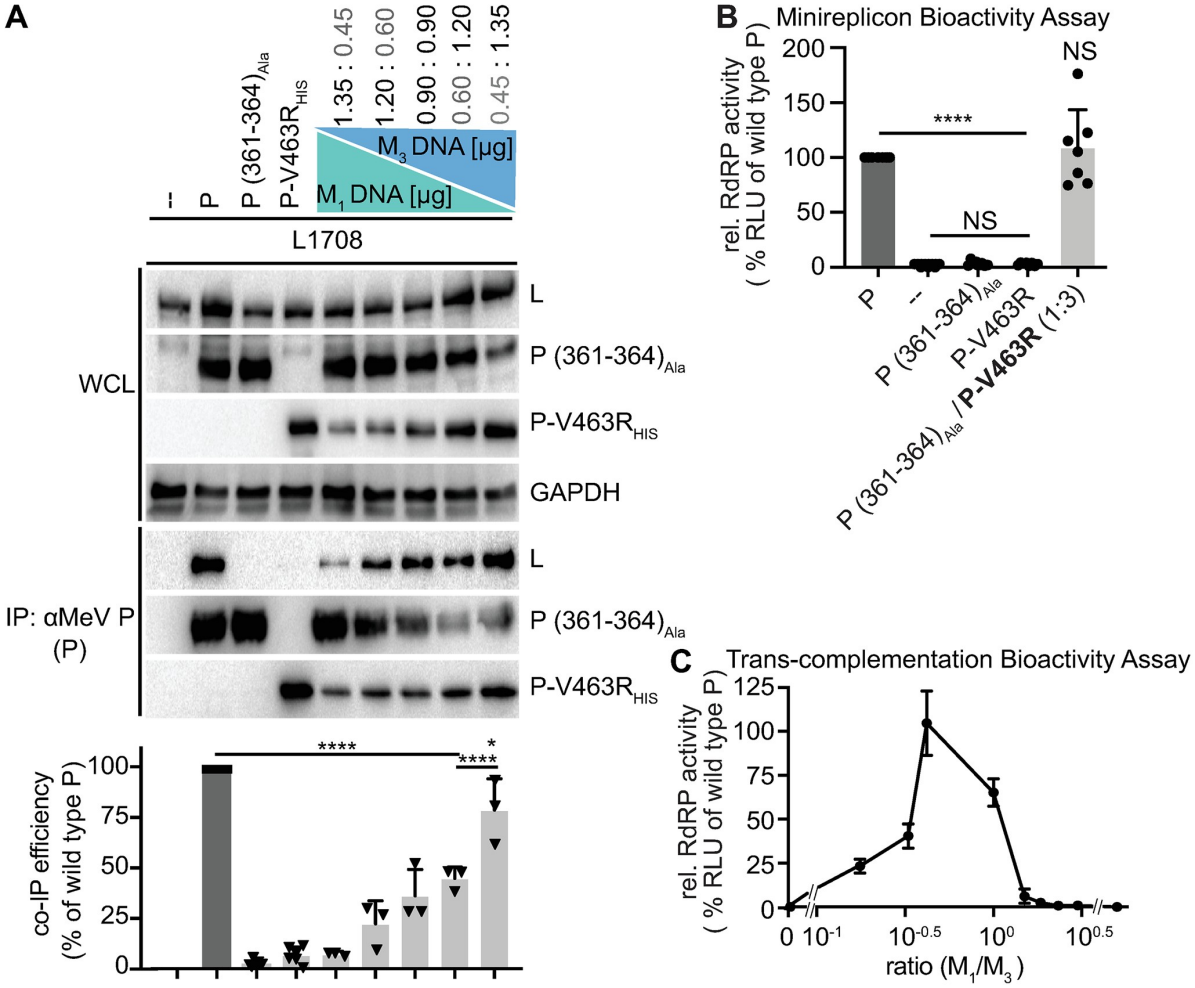
L-binding null-mutants in P OD C-terminal microdomain and P-XD efficiently trans-complement. **A)** Graphic depiction of P (361–364)_Ala_ / P-V463R trans-complementation ratios tested and P:L interaction analysis of the different trans-complementation pairs. A HIS-tagged version of the P-V463D mutant was used to enable differential immunoprecipitation, detection and quantitative analysis otherwise as in Fig 1C (n ≥ 3). **B)** Minireplicon assay of candidate trans-complementation P mutants alone and co-expressed at 1:3 relative ratio (n ≥ 3). **C)** Trans-complementation RdRP activity profile of the pair shown in (A) and (B), analyzed in minireplicon assays over the specified relative ratio range. Symbols show means of biological repeats ± SD (n = 3). All statistical analyses and symbols as detailed in Fig 1.

Having identified two discrete P microdomains that are required for interaction with L and well-conserved across several major pathogens in the paramyxovirus family (S6 Fig), we explored whether these domains are functionally distinct. Co-expression of P (361–364)_Ala_ and P-V463R, each by itself unable to co-IP L, restored physical interaction with L (Fig 5A). Wild type P-like binding efficiency was observed in the presence of the highest relative excess of P-V463R tested. When applied to minireplicon assays, we found that successful trans-complementation of L binding capacity extended to bioactivity of mixed P tetramers, remarkably resulting in RdRP activity equivalent to that observed with wild type P when cells received P (361–364)_Ala_ and P-V463R-encoding plasmid DNA in approximately 1:3-relative ratio (Fig 5B). Trans-complementation profiles over a wide plasmid ratio range corroborated this result, revealing a steep, asymmetric bell curve with a wild type P-equivalent RdRP bioactivity peak at a relative plasmid DNA ratio of 1:3 (P (361–364)_Ala_:P-V463R) (Fig 5C). We conclude that at least two structurally and functionally distinct P microdomains are engaged in productive P-to-L binding. Wild type P-like bioactivity in the presence of an excess of P-V463R in mixed P tetramers corroborates our earlier observation that designating L binding-competence to a single XD per P tetramer boosts bioactivity, thus compensating for the negative effect associated with the presence of even one P (361–364)_Ala_ monomer.

**Fig 6:**
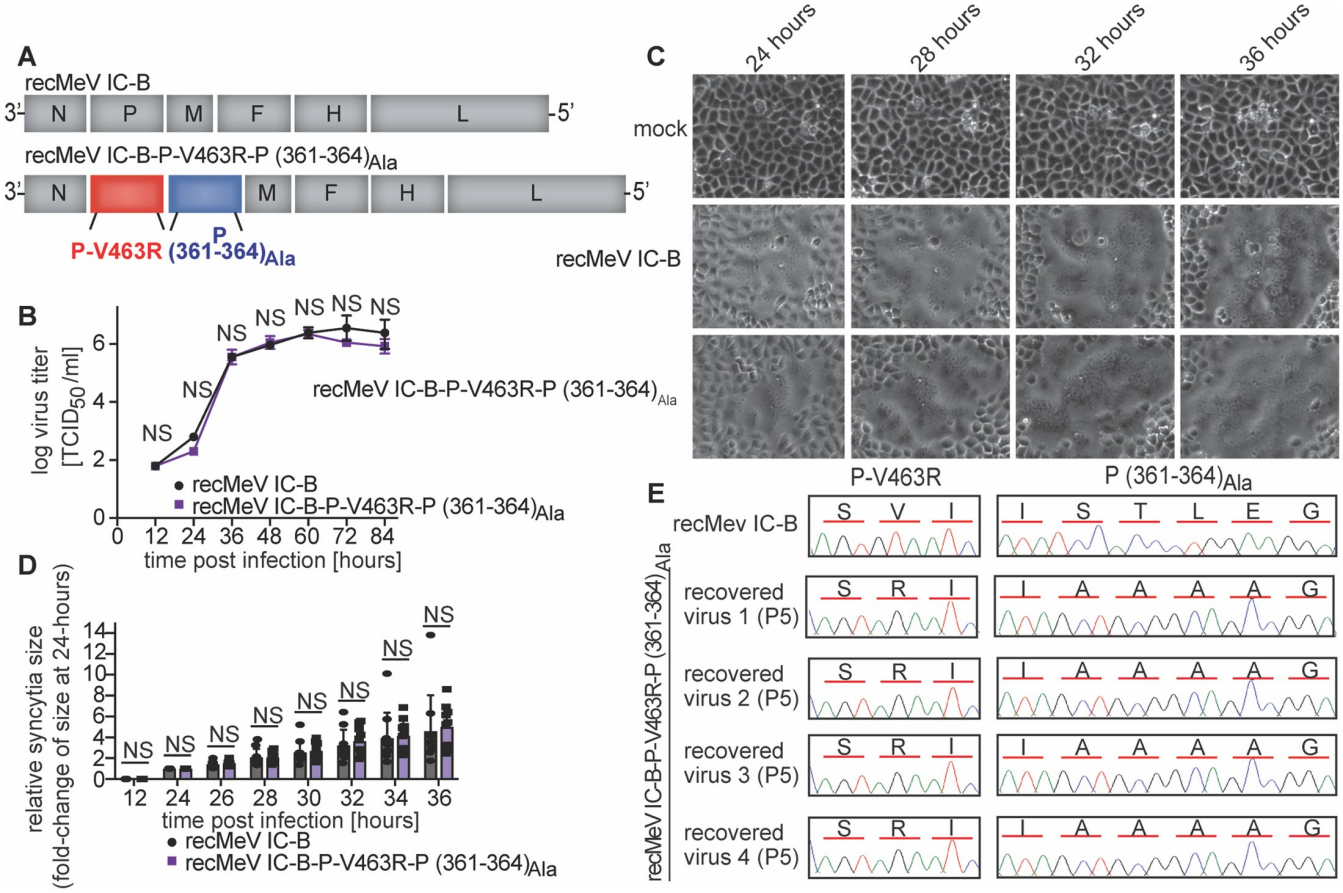
**A)** Graphic genome representations of standard MeV (strain IC-B) and trans-complementation candidate. The relative positions of the P-V463R (red) and P (361–364)_Ala_ (blue) genes are highlighted. **B)** Viral growth curves after recovery of parent recMeV IC-B and trans-complemented recMeV IC-B P-V463R-P (361–364)_Ala_. Symbols represent mean titers of biological repeats ± SD (n = 3). Statistical analysis through two-way ANOVA with Sidak’s post-hoc comparison tests. **C)** Microphotograph time-courses of uninfected Vero-hSLAM cells (mock) or cells infected with recMeV IC-B or recMeV IC-B P-V463R-P (361–364)_Ala_, taken in 4-hour intervals from 24–36 hours after infection. Each series shows a specific area, monitored over time. **D)** Quantitation of CPE kinetics after infection of cells with standard and trans-complemented recMeV. Syncytia sizes in 10 distinct areas/virus were quantitated automatically using a high-content imager, and are each expressed as fold-change increase in syncytia size (in μm^2^ area covered) relative to syncytia size at the same plate coordinates at 24 hours pI. Columns represent means of experimentally observed values ± SD, symbols show individual repeats (n = 10); NS not significant. **E)** Chromatograms of P sequences at mutated areas of recovered recMeV IC-B and recMeV IC-B P-V463R-P (361–364)_Ala_ after five serial passages on Vero-hSLAM cells. Encoded amino acids are specified above the chromatograms.

Minireplicon assays are highly informative for functional dissection of RdRP activities, but the physiological relevance of the results can be compromised by the shortcoming that only a subset of the multiple RdRP activities required for successful virus replication is examined [28]. To test whether trans-complementation restores the complete functional range of MeV RdRPs, we engineered a recMeV genome in which the wild type P gene was replaced with a tandem arrangement of genes encoding P-V463R and P (361–364)_Ala_, respectively (Fig 6A). P-V463R was intentionally placed upstream of P (361–364)_Ala_ to capitalize on the *Mononegavirales* transcription gradient [49] and ensure that L binding-deficient P-V463R would be present in relative abundance over P (361–364)_Ala_ in infected cells. Corresponding recMeV P-V463R-P (361–364)_Ala_ was recovered successfully, replicated as efficiently as the genetic parent virus (Fig 6B) and displayed equivalent cytopathic effect (CPE) size (Fig 6C) and CPE kinetics (Fig 6D) in cell culture. Sanger sequencing of both P genes after five consecutive passages of four independently recovered recombinants confirmed the integrity and genetic stability of both P mutations in replicating virus (Fig 6E). Like all paramyxovirus RdRPs, MeV polymerase is error-prone and mutations advantageous for viral fitness become genetically fixed rapidly [50]. The clean chromatograms at both mutation sites in all recMeV examined indicate a remarkable lack of selective pressure to acquire revertant or compensatory mutations. This observation was consistent with the parent recMeV-like growth profiles of the double mutant virus and underscores that all P bioactivities required for unimpaired virus replication are restored through trans-complementation.

### OD proximal and connector region residues reside in the same functional group

**Fig 7:**
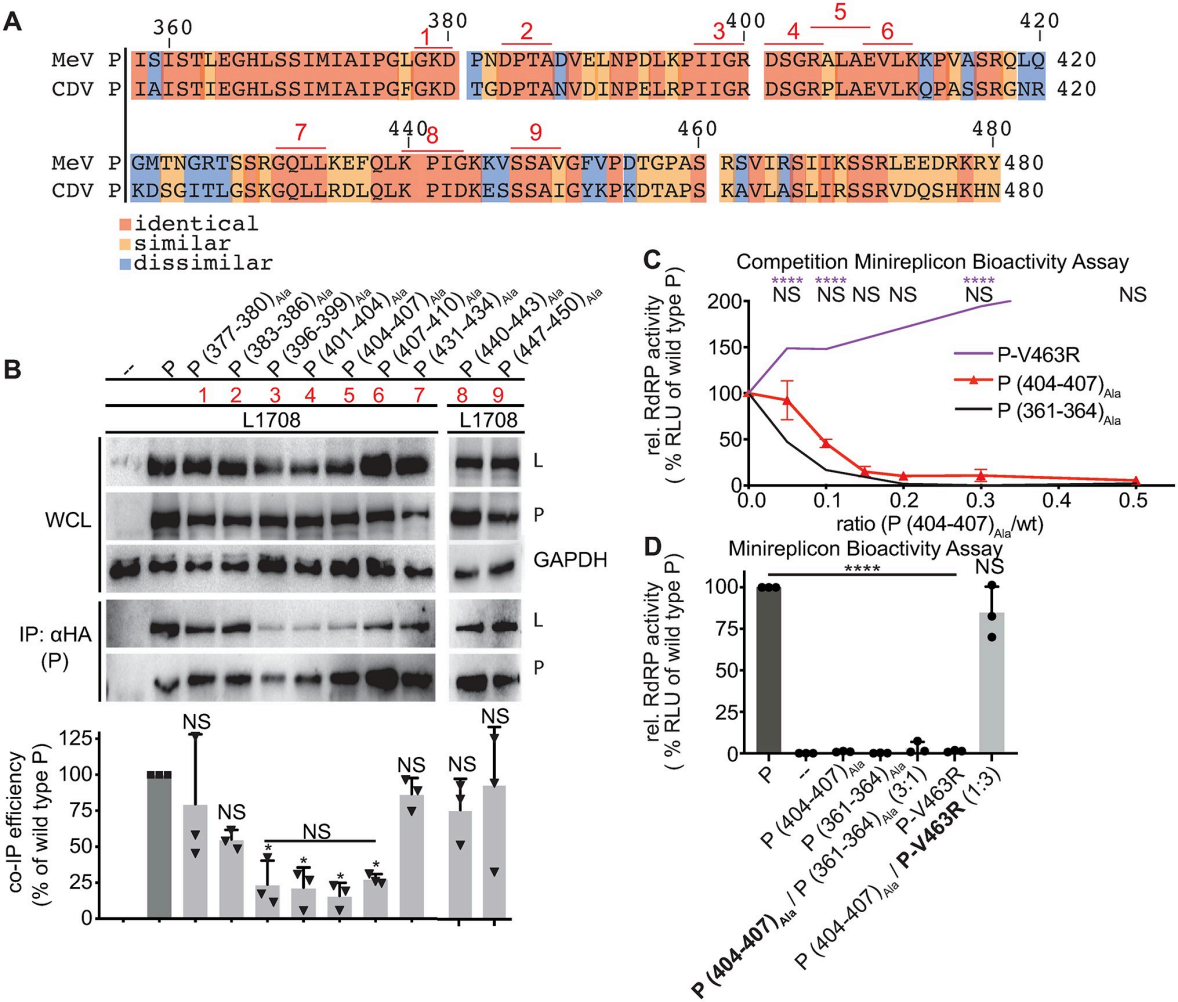
Residues in the connector region between P position 377 and 458 trans-complement P-XD mutations but are functionally linked to the OD C-terminal microdomain. **A)** Alignment of MeV and CDV P sequences between residues 358 and 480. Conserved patches (≥3 identical residues) are underlined and numbered consecutively. **B)** P:L interaction analysis of P mutants with alanine substitutions of residues in conserved patches identified in (A; red numbers). Detection and quantitative analysis as in Fig 1C (n = 3). **C)** Minireplicon competition experiment between P (404–407)_Ala_ and wt P, set-up as in Fig 2D and 2E. Symbols show means of relative RdRP activity ± SD, three biological repeats. Purple and black lines show competition profiles respectively between P-V463R (Fig 4G) and P (361–364)_Ala_ (Fig 2E). Statistical analysis with two-way ANOVA and Sidak’s post-hoc multiple comparison test. **D)** Trans-complementation minireplicon assays of P (404–407)_Ala_ with P (361–364)_Ala_ and P-V463R, respectively, relative ratios of P-encoding plasmid DNA transfected as specified. Columns represent mean relative RdRP activities ± SD, symbols show biological repeats (n = 3). Statistical analyses in (B) and (D) and symbols as in Fig 1.

To scan the approximately 80-residue connector region between the P OD C-terminus and P-XD for amino acids potentially contributing to L binding, we explored heterotypic interactions between MeV and very closely related canine distemper virus (CDV) P and L. Both viruses share considerable protein sequence homology and identity, and heterotypic co-IPs confirmed that MeV P associates with MeV or CDV L with equal efficiency (S7 Fig). Heterotypic interaction suggests that any residue in the connector region contributing to P-to-L binding should be conserved between both viruses. We identified nine distinct microdomains of three to four consecutive identical amino acids in sequence alignments of the MeV and CDV P connector regions (Fig 7A) and subjected each individually to alanine scanning mutagenesis.

Co-IPs with the resulting MeV P mutants highlighted a stretch of four consecutive conserved patches in the connector domain, spanning P residue 396 to 410, that significantly impaired P-to-L interaction when mutated (Fig 7B). A local co-precipitation minimum was reached by the P (404–407)_Ala_ mutant. However, all mutants tested with alanine substitutions in the connector domain, including those capable of physical interaction with L, significantly reduced P bioactivity in minireplicon assays (S8 Fig). When increasing amounts of P (404–407)_Ala_ were co-transfected with wild type P in minireplicon competition assays, bioactivity profiles markedly differed from those of the P-XD mutants but closely resembled the dominant-negative P (361–364)_Ala_ (Fig 7C). Trans-complementation of P (404–407)_Ala_ was likewise successful with P-V463R but not with P (361–364)_Ala_ (Fig 7D), placing the P OD C-terminal residues and connector region in the same complementation group, each functionally distinct from the L binding face of P-XD.

### P-XD is a central regulator of the dynamic interplay between polymerase and encapsidated template

**Fig 8:**
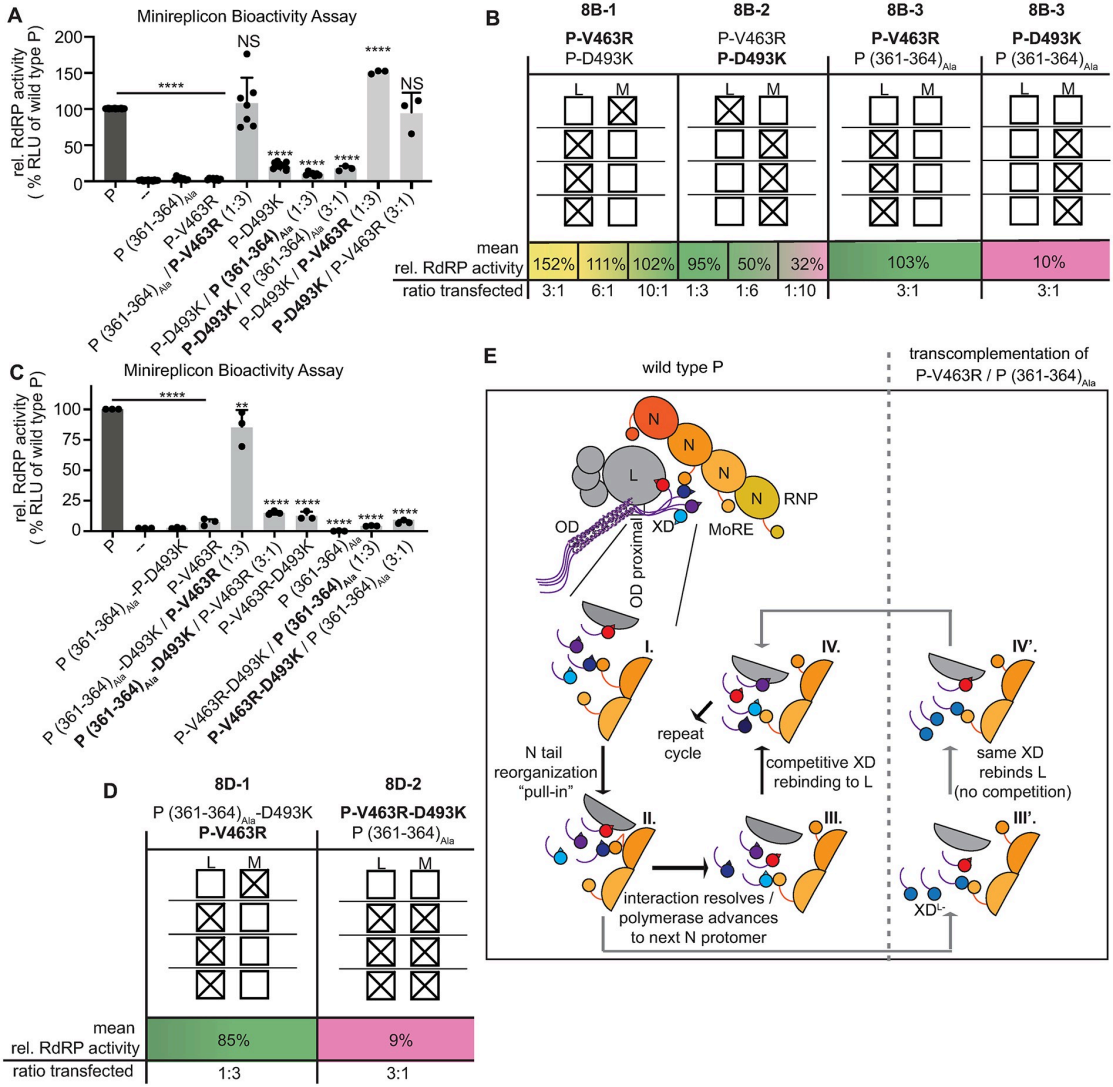
2-way and 3-way trans-complementations to probe stoichiometric requirements of the different P-XD functionalities in bioactive RdRP complexes. **A, C)** Trans-complementation minireplicon assays of P single and double mutants as specified. Relative ratios of P-encoding plasmid DNA transfected are indicated. Columns represent mean relative RdRP activities, symbols show individual biological repeats ± SD (n ≥ 3). Statistical analyses through one-way ANOVA and Tukey’s multiple comparison test, symbols as detailed in Fig 1. **B, D)** Schematic overview of the predominant P tetramer populations of selected trans-complementation pairs from (A) and (C) as specified. P-encoding plasmid DNA ratios transfected are indicated and mean relative RdRP activities are color-coded (green, wild type P-like activity; yellow, hyper-active; magenta, inactive). Boxes represent dominant XDs composition of trans-complemented mixed P tetramers. Open boxes indicate L (L) or MoRE (M) binding-competence, crossed boxes denote the presence of loss-of-function substitutions for the respective binding activity. **E)** Mechanistic summary model of P interaction with L and P-XD-mediated regulation of RdRP negotiation of the N-encapsidated template. Individual XDs of the P tetramer are differentiated by color, Ncore and MoREs are shown in orange. Roman numerals in close-up inserts depict specific interactions identified through trans-complementations and are discussed in the text. A model for RdRP activity boosting by the signature trans-complementation pair P-V463R / P (361–364)_Ala_ is shown on the right.

To query whether the same P-XD monomer in a P tetramer must be both L and MoRE binding-competent to support RdRP activity, we examined trans-complementation of the P-D493K mutation abrogating P-XD-to-MoRE binding with P-V463R blocking P-XD-to-L interaction at different relative ratios. Efficient trans-complementation occurred between MoRE-binding impaired (L^+^ MoRE^−^) and L-binding defective (L^−^MoRE^+^) P-XDs (Fig 8A). Albeit trans-complementation efficiency was reduced at each of the ratio extremes (1:10 and 10:1, respectively)–presumably reflecting accumulation of a large proportion of bio-inactive homotypic mutant tetramers under these conditions—appreciable RdRP activity remained (Fig 8B). We then attempted trans-complementation between P-D493K and P (361–364)_Ala_. Since our mathematical modeling has demonstrated that at most one P (361–364)_Ala_ monomer can be present in a bioactive P tetramer, this mutant enabled us to probe the stoichiometric requirements of the different P-XD functionalities. In contrast to our successful earlier trans-complementation of (L^−^MoRE^+^) P-XDs with P (361–364)_Ala_, the combination of (L^+^ MoRE^−^) P-XDs with P (361–364)_Ala_ did not restore RdRP activity. These results indicated that the presence of one MoRE binding-competent or one L binding-competent XD per P tetramer is sufficient for RdRP activity, but these competencies must not reside on the same P-XD if the other three P-XDs are unable to interact with MoRE (Fig 8B).

Three-way trans-complementation studies, in which we paired each of the three functional groups tested in all possible combinations reinforced this conclusion, since P-V463R efficiently trans-complemented with a P-D493K (361–364)_Ala_ double mutant, while the combination of P-V463R-D493K with P (361–364)_Ala_ was bio-inactive (Fig 8C and 8D). The presence of only one each L and MoRE binding-competent XD in P tetramers is therefore necessary and sufficient to achieve approximately wild type P-like bioactivity, but both binding partners cannot be simultaneously engaged by the same P-XD monomer. Interaction of a P-XD with L takes priority over MoRE binding when the number of fully functional XDs present in mixed P tetramers is restricted. These data reveal P-XD as a regulatory element of the highly dynamic and multi-faceted protein-protein interplay between RdRP and the RNP template, placing it at the center of a mechanistic model of polymerase advancement.

## Discussion

This study demonstrates that distinct microdomains in MeV P are involved in efficient P hetero-oligomerization with L. We favor the view that these microdomains engage L through direct protein-protein contacts, although more indirect associations involving, for instance, host-derived adapter proteins cannot be excluded. Here, the term P:L interface will be used to refer to both direct and multi-component interactions between the P and L proteins. Based on a combination of biochemical and functional assays, we have pioneered a trans-complementation system to probe the stoichiometric requirements for productive P:L interactions. While trans-complementations were carried out in minireplicon assays, a recombinant MeV carrying a P trans-complementation pair in two tandem open reading frames could be recovered readily, efficiently proliferated with standard MeV-like growth profile, and was genetically stable over multiple passages. The absence of compensatory or reversion mutations, in particular, serves as a compelling indicator that the mutant recMeV does not experience strong selective pressure. These results confirm that trans-complementation restored all RdRP activities required for successful paramyxovirus replication. Thus assured of the physiological relevance of the strategy, the assay has advanced our mechanistic understanding of how paramyxovirus RdRp negotiates the encapsidated template in four areas:

1. The P:L interface is bipartite. P monomers were unable to interact with L, but replacement of P-OD with yeast GCN4 was tolerated, demonstrating that P tetramerization is essential for L binding. In contrast to previous suggestions [23], however, residues located in the P OD core section are not part of the physical interface. Rather, a 17-residue stretch at the C-terminal end of P OD mediates L binding after extension of the helical coiled-coil up to residue 377. This conclusion is supported by the natural continuation of the heptad repeat patterns of P OD through to position 377, complete abrogation of the ability of L to co-IP with P after insertion of alanine substitutions in this microdomain, and the dominant-negative competition profile of P (361–364)_Ala_ with wild-type P. It furthermore is consistent with a very recent study demonstrating that the coiled-coil OD of MeV P is required for viral gene expression [9]. Mathematical description of the experimentally determined data revealed that in the presence of both P (361–364)_Ala_ and wild type P, only mixed P tetramers harboring precisely one copy of this mutant are bioactive. We have thus shown that three wild type P monomers are able to structurally control one mutant and form proper tertiary assemblies, but two or more P (361–364)_Ala_ subunits in a P tetramer cannot be contained.
  Downstream of the P OD proximal section, we identified a conserved second microdomain centered around P residues 404–410 that was a major determinant for the efficiency of P-to-L binding. Changes in this connector region likewise had a dominant-negative effect on P bioactivity, suggesting that the section between P OD and P-XD very likely does not exist monomeric in P complexes, but engages in a tertiary assembly. Trans-complementation revealed that the P-OD proximal and connector sections reside in the same functional groups, raising the possibility of an extended interface with L or, if only one of these regions engages in physical contact with L, interference of mutations in the other microdomain with proper assembly of the actual contact zone.
  Remarkably, we identified specific residues located on two different α-helices of P-XD that are essential for P-to-L binding. These residues map to a microdomain on a previously unappreciated face of the P-XD triangular prism that is distinct from the side binding MoRE. Importantly, this microdomain resides in a separate functional group from the P OD proximal and connector regions, and mutations destroying this newly discovered L-binding face of P-XD were recessive in competition studies with wild type P. These results indicate that the P-XD interaction with L is mediated by a single XD subunit of the P tetramer, and that at least two distinct binding sites for different P microdomains exist on L, the P oligomerization domain-proximal tetrameric regions (tetrameric feet) and the P-XD (monomeric head) (Fig 8E). Neither site was able to form biochemically appreciable complexes with L in isolation, indicating moderate binding affinities of both interfaces. The formation of stable P-L hetero-oligomers appears to require the avidity gain arising from at least two independent contact sites.
2. Only one each of the four XDs per P tetramer must be L or MoRE binding-competent (Fig 8B-1, 8B-2 and 8D-1). By introducing point mutations in P-XD to the trans-complementation assay that selectively disrupt MoRE or L binding, we demonstrated that a single MoRE binding-competent P-XD is sufficient for wild type P-like RdRp bioactivity (Fig 8B-2). This finding is paradigm-shifting since prevailing models assign cartwheeling function to the four XDs of a P tetramer [29, 30], in which the polymerase supposedly advances along the RNP template through iterative formation and release of P-XD contacts with MoRE, ensuring that at all times at least one intact P-XD-MoRE interface is present. Our results demonstrate that no stringent requirement exists and no P-XD-mediated cartwheeling needs to occur. This conclusion is also consistent with our earlier observation that RNPs formed by N mutants with tail deletions encompassing the C-terminal 86 amino acids that include MoRE are partially bioactive [28].
3. P-XD interaction with L and MoRE is mutually exclusive, and L-binding of a given P-XD supersedes MoRE-binding (Fig 8D-2, 8B-3 and 8D-4). These conclusions are based on the central premise revealed by the mathematical regression analysis of the P (361–364)_Ala_ competition profile with wild type P, that at most one P (361–364)_Ala_ monomer can be present in bioactive mixed P tetramers. Employing the P (361–364)_Ala_ mutant as a tool to control the stoichiometry of trans-complemented P tetramers, we noted that although only one each of the four P-XDs must be L or MoRE binding-competent, combining these competencies on only one P-XD per tetramer completely abrogates polymerase activity (Fig 8D-2). Presumably, steric hindrance prevents that the same P-XD simultaneously engages both MoRE and L. Importantly, the presence of only one (L^+^ MoRE^+^) P-XD and three (L^−^MoRE^+^) P-XDs in a mixed P tetramer was sufficient to restore wild-type P like polymerase activity (Fig 8B-3), but the inverse constellation (one (L^+^ MoRE^+^) P-XD and three (L^+^ MoRE^−^) P-XDs) was entirely bio-inactive (Fig 8B-4). This complementation phenotype revealed dominance of the P-XD interaction with L over MoRE, yet raised the question of why in the constellation depicted in Fig 8B-4 75% of the P-L hetero-oligomers (those in which one of the (L^+^ MoRE^−^) P-XDs happens to contact L) did not remain bioactive.
4. During RNA synthesis, P-XD iteratively separates from and reengages with L (Fig 8B-1 and 8B-4). In all trans-complementation constellations that assigned L-binding competence to only one P-XD, we consistently noted a boost in RdRP bioactivity compared to wild type P tetramers. This unexpected phenotype can be best appreciated in the Fig 8B-1 setting, but is equally present in the Fig 8B-3 and 8D-1 constellations, since addition of only one P-(361–364)_Ala_ monomer to otherwise wild type P tetramers reduced bioactivity by approximately 40% according to our regression model. Consequently, wild type P-like 100% bioactivity seen in the Fig 8B-3 and 8D-1 constellations reflects a near-perfect additive effect of the activity booster resulting from P-XD assignment and penalty associated with presence of a P-(361–364)_Ala_ monomer. Our competition studies of the P-V463R mutant with wild type P returned even higher peak RdRP values reaching 250% of reference bioactivity.

What could be the mechanistic basis for a boost in RdRP activity when only one P-XD subunit is able to interact with L? A previous study considered that negotiation of the encapsidated template by paramyxovirus polymerases might depend on continuous structural rearrangements within the RdRP complex, possibly including the repetitive dissolution and reassembly of P:L interaction [2]. Based on our identification of a bipartite P:L interface and the trans-complementation data, we propose that the newly discovered P-XD:L interface periodically resolves and reforms. In wild type P tetramers, naturally all four P-XDs are L binding-competent. Iterative separation of P-XD from L creates opportunity for competition between the individual P-XDs to reengage with L and/or need for rearrangement within the P-L hetero-oligomer every time a physically different P-XD than before is successful. Delegating L binding to a designated P-XD monomer as in our mixed P tetramers eliminates internal competition and associated rearrangements of the complex, unleashing maximal polymerase processivity. This conclusion is consistent with the mathematical description of our experimental data and provides ready explanations for why a bipartite P:L interface may have evolved—two distinct contact zones allow temporary separation of only the P-XD:L interface without presumably catastrophic full separation of P from L—and why the trans-complementation pair depicted in Fig 8B-4 is bio-inactive. If P-XD indeed transiently separates from and rebinds to L as the polymerase advances along the template, the single (L^+^ MoRE^+^) P-XD monomer present in every RdRP complex that can potentially initiate RNA synthesis in this constellation will eventually engage L. Because no other MoRE binding-competent P-XDs are present in that P tetramer, this event appears to create a dead-end situation, presumably resembling that experienced by the trans-complementation pair represented by Fig 8D-2.

We propose a 4-step model to describe the dynamic interplay between P, L, and the RNP (Fig 8E). The tetrameric base of P remains latched to L at all times, while the P-XD head reiteratively taps L. In the case of wild type P, one P-XD forms a temporary complex with L while another P-XD binds MoRE (stage I). MoRE engagement is thought to induce reorganization of N-tail [51], moving it closer to the core of the RNP helix (stage II). Possibly this change in microenvironment of the binding partners may destabilize the interfaces, resulting in temporary separation of both P-XD:MoRE and P-XD:L, which enables advancement of the polymerase to the next N protomer (stage III). One of the four P-XDs then rebinds to L and another engages the MoRE of the new N protomer (stage IV), followed by a repeat of the cycle. Assignment of L-binding competence to a specific P-XD does not fundamentally change the process, but P-XD competition for the L binding site as well as potential rearrangements within the complex when a separate P-XD moiety was successful are eliminated, since at every cycle the same P-XD monomer rebinds to L (stage III’ to IV’). The remaining two P-XDs not fully depicted in Fig 8E may or may not engage other MoREs in the vicinity. However, these interactions are not essential for polymerase activity, if they occur. This mechanistic model assigns a fundamental role in regulating polymerase activity to the paramyxovirus P protein and identifies a novel principle—reiterative separation and restoration of P-XD interaction with L—that kinetically regulates RdRP negotiation of the encapsidated RNA template.

## Materials and methods

### Cell culture

Baby hamster kidney cells (C-13; ATCC) stably expressing T7 polymerase (BSR-T7/5), African green monkey kidney epithelial (Vero) cells (CCK-81; ATCC), and Vero cells stably expressing human signaling lymphocytic activation molecule (Vero-hSLAM) were maintained in Dulbecco’s modified Eagle’s medium (DMEM) supplemented with 7.5% fetal bovine serum at 37°C and 5% CO_2_. The stable cell lines were incubated in the presence of G418 (Thermo-Fisher) (100 μg/ml) at every fifth passage. Cells were transiently transfected using GeneJuice (Novagen) according to the manufacturer’s instructions.

### Molecular biology

Codon-optimized open reading frames encoding MeV IC-B-derived L, L_1708_, and P were synthesized *in vitro* (GeneWiz). The PΔOD variant lacking residues 303–360 was generated through PCR amplification and religation at an added HindIII site. Yeast-derived GCN4 tetramerization domain was PCR amplified from a previously generated template [52] and inserted using the HindIII site. All alanine substitutions and amino acid changes were performed by site-directed PCR mutagenesis using the QuikChange protocol (Stratagene). C-terminal truncations were performed by PCR mutagenesis and subsequent religation at an added AgeI site. Plasmids encoding the MeV minireplicon luciferase reporter, non-optimized MeV IC-B N, IC-B P, and IC-B L under T7 promoter control, and a full-length cDNA copy of the MeV IC-B genome were previously described [47]. A full-length cDNA of recMeV IC-B expressing P-V463R and P (361–364)_Ala_ in tandem was generated based on non-codon optimized mutant versions of IC-B P using the NEBuilder HiFi DNA Assembly kit, introducing and artificial intergenic sequence (IGS) between the two P copies. The assembled recMeV IC-B P-V463R-N-IGS-P (361–364)_Ala_ cassette was transferred to the full-length genomic plasmid between the N-P and P-M intergenic junctions using existing XbaI and SalI restriction sites. Mutagenesis success and the integrity of all PCR-amplified nucleic acids was confirmed through Sanger sequencing.

### SDS-PAGE, immunoblotting and densitometric quantitations

Cells were transfected in a 6-well plate format (5 x 10^5^ cells/well) with 1 μg of plasmid DNA encoding codon-optimized MeV L_1708_ with a C-terminal FLAG epitope tag and 1.8 μg of plasmid DNA encoding MeV P or P mutants, each with a C-terminal HA epitope tag. After 36 hours, cells were washed two times with phosphate buffered saline (PBS) and lysed chemically (50 mM HEPES (pH 7.2), 300 mM NaCl, 1.0 mM EDTA, 1% Triton X-100, protease inhibitors (Roche)). Cleared (10-minute centrifugation at 12,000 rpm, 4°C) lysates were mixed with 5×urea buffer (200 mM Tris/Cl [pH 6.8], 8 M urea, 5% SDS, 0.1 mM EDTA, 0.03% bromophenol blue, 1.5% dithiothreitol). Samples were incubated for 30 minutes at 50°C and separated on 8% SDS-PAGE gels, tank-blotted on polyvinylidene difluoride (PVDF) membranes (Millipore), and subjected to chemiluminescence detection using specific antibodies directed against the FLAG (M2; Sigma) or HA (16B12; Abcam) epitopes, MeV N (clone 83KKII; Millipore Sigma), or against cellular glyceraldehyde-3-phosphate dehydrogenase (GAPDH; 6C; Ambion) as specified. Immunoblots were developed using a ChemiDoc digital imaging system (Bio-Rad) for image visualization. Densitometry was carried out on non-saturated images with global background correction. A full set of positive (wild type P) and negative (equivalent amount of vector DNA replacing P-encoding plasmid DNA) controls were included on each immunoblot, and no normalizations across different blots were conducted.

### Native-PAGE

Cells transfected with 1.0 μg MeV P or P mutant-encoding plasmid DNA were harvested after 36 hours as specified above. Cleared lysates were mixed with 1 μl G-250 sample additive (Invitrogen) and 4×Native-PAGE sample buffer (Invitrogen). Electrophoretic gel fractionation was carried out using 3–12% Bis-Tris gradient gels (Invitrogen) and NativePAGE running buffer. Immunoblotting and detection were performed as outlined above.

### Co-immunoprecipitation

Cells (5 × 10^5^ cells/well) were transfected with MeV L_1708_ and P or P mutant-encoding plasmid DNA as detailed above. Cells were lysed 24 hours after transfection and cleared lysates incubated with specific antibodies directed against HA or HIS epitopes (HIS.H8; Invitrogen; only for immunodetection after trans-complementation; Fig 5A), or against MeV N (only for immunoprecipitations in Fig 4D) at 4°C, followed by precipitation of immunocomplexes with immobilized protein G (Pierce) in 50 μl bed volume at 4°C. G-protein bound protein samples were washed twice each in cold lysis buffer and PBS, each wash with 20 bed volume equivalents (1 ml), followed by resuspension in 5x urea buffer. Denatured samples were subjected to SDS-PAGE analysis using 8% homogenous gels followed by immunoblotting and detection using specific antibodies directed respectively against the FLAG, HA, or HIS epitopes as described. To calculate relative co-immunoprecipitation efficiencies, densitometric quantitations of co-precipitated L were normalized for those of L co-precipitated by standard P. This approach is based on the rationale that although L turnover rates are increased in the absence of P or presence of L binding-incompetent P mutants, synthesis rates of plasmid-encoded L is independent of P and standard and mutant P have therefore equal initial opportunity to productively interact with nascent L polypeptides.

### Minireplicon reporter assay

BSR-T7/5 cells (5,000 cells/well in a 96-well plate format) were transfected in nine technical replicates per condition assessed with plasmids encoding MeV IC-B L (0.02 μg), IC-B N (0.016 μg), the MeV luciferase replicon reporter (0.044 μg), and either IC-B P or P mutants as specified (0.02 μg unless stated otherwise in Figure captions). In all experiments that involved transfecting variable amounts of mutant and wild type P-encoding plasmid DNA ratios, empty vector (pUC-19) DNA was added in the appropriate amounts to ensure that all transfection reactions received the same total amount of DNA. Firefly luciferase activities were determined 24 hours post-transfection in a Synergy H1 microplate reader (BioTek), using Bright-Glo luciferase substrate (Promega) directly added to the wells and signal detection after a 1 to 2-minute stabilization period. Relative RdRP activities, expressed as percentages of that observed in the presence of wild type P, were determined according to the formula % rel. activity = (signal_sample_-signal_min_)/(signal_max_-signal_min_) × 100, with signal_max_ corresponding to cells having received wild type P and signal_min_ corresponding to cells having received equal amounts of pUC-19 in place of P-encoding plasmid. Results calculated for each biological repeat represent the means of the nine technical repeats, and each condition (P mutant, competition or trans-complementation setting) was assessed in at least three biological repeats.

### Recovery of recombinant MeV

recMeV were recovered by transfection of BSR-T7/5 cells with full-length antigenomic plasmid (1.25 μg) and the plasmids encoding IC-B N (0.42 μg), IC-B-P (0.54 μg), and IC-B-L (0.55 μg). Transfected cells were overlaid after 48 hours onto Vero-hSLAM cells. Emerging infectious centers were individually transferred to and then passaged twice on Vero-hSLAM cells, followed by whole RNA extraction from infected cells (RNeasy kit; Qiagen), first strand synthesis using random hexamer primers and Superscript III reverse transcriptase (Invitrogen), PCR amplification of synthesized cDNAs using appropriate primers, and Sanger sequencing. Titers of MeV stocks were determined through TCID_50_ titration on Vero-hSLAM cells as described [53].

### Viral growth kinetics

Vero-hSLAM cells were plated in 12-well format (1.0×10^5^ cells/ well) and infected with recovered viruses, either recMeV IC-B or recMeV IC-B P-V463R-P (361–364)_Ala_ at a multiplicity of infection (MOI) 0.01 TCID_50_ units per cell for 1-hour, followed by replacing the inoculum with growth media. To ensure accurate inoculum concentrations, virus stocks were pre-diluted to approximately 5,000 TCID_50_/ml and inoculum titers validated through TCID_50_ titration. Cell-associated viral particles were harvested in 12-hours intervals through scraping of cells in serum-free DMEM, two consecutive freeze/thaw cycles, and clearance centrifugation (2,000 rpm, 4°C). Viral titers in cleared samples were determined through TCID_50_ titration.

### Photometric CPE quantitation

Vero-hSLAM cells were plated in a 12-well format (1.0×10^5^ cells/well) and infected with either recMeV IC-B or recMeV IC-B P-V463R-P (361–364)_Ala_ at a MOI of 0.01 TCID_50_/cell for one hour, then inoculum was replaced with growth media. Infected cells were imaged in 2-hour increments over a period of 36 hours post-infection with a Cytation5 automated high content imager with phase contrast microscopy capacity (BioTek). The increase in μm^2^ area size covered by individual infectious centers first detected at 24 hours after infection was quantified over time using the Gen5 microplate software program (Ver. 3.05.11) (BioTek). A maximum of four syncytia/well were followed, quantitations are based on 10 distinct syncytia/virus analyzed. CPE kinetics are expressed as fold-change of area size relative to the 24-hour after infection timepoint.

### Mathematical models of bioactivity

Based on behavior in native-PAGE, mutant and wild type P co-expressed in the same cell were given equal probability to tetramerize and the mutant occupation of one P monomer was treated independent of the other three monomers. The ratio between mutant (M_x_) and wild type (wt) P was denoted as ρ. Adapting a previous approach to a comparable problem [54], the probabilities of the formation of tetramers with different compositions were calculated as follows:

$$P_{4wt}\;=\;{\left(\frac{1}{1+\rho }\right)}^{4},$$

$$P_{3wt1M}\;=\;4{\left(\frac{1}{1+\rho }\right)}^{3}{\left(\frac{\rho }{1+\rho }\right)}^{1},$$

$$P_{2wt2M}\;=\;{6(\frac{1}{1+\rho })}^{2}{\left(\frac{\rho }{1+\rho }\right)}^{2},$$

$$P_{1wt3M}\;=\;{4(\frac{1}{1+\rho })}^{1}{\left(\frac{\rho }{1+\rho }\right)}^{3},$$

$$P_{4M}\;=\;{\left(\frac{\rho }{1+\rho }\right)}^{4}.$$

A number of interactions of host proteins with MeV P and N have been proposed [55], but very limited insight into this complex interactome precludes integration into rational modeling. We therefore applied a deconstruction approach, concentrating on individual point mutations affecting specific interactions between P-XD and L or N-MoRE, respectively. At any given mutant to wild type P ratio ρ, all five different P tetramer compositions will be present in the system (illustrated in Fig 2D). Two hypotheses regarding the bioactivities of these tetramer species were examined. In both models, the α-values reflect the individual contribution of each of the different P tetramer assemblies to overall bioactivity, with consideration of the relative abundance of each assembly in the system.

1. Linear combination; all tetramers with at least one wild type P monomers are bioactive and their contributions to RdRP bioactivity (A) are linearly additive:
  $$A\;=\;\sum_{i\;=\;1}^{4}{\alpha_{i}P}_{iwt}\;=\;{\alpha_{0}P_{0wt4M}+\alpha }_{1}P_{1wt3M}+\alpha_{2}P_{2wt2M}+\alpha_{3}P_{3wt1M}+\alpha_{4}P_{4wt0M}$$
  Assumptions made: all-mutant P tetramers (P_0wt4M_) are bio-inactive (Figs 2C and 4E, S4 Fig), *α*_0_ = 0. Therefore, only four weights needed to be fitted; positive weights reflect a positive and negative weights negative contributions to overall bioactivity; tetramer species with a weight of 0 are bio-inactive.
2. Non-linear pairwise P-XD competition; in addition to each tetramer species potentially contributing to bioactivity, a possible impact of competition for binding sites was considered:
  A=XDbindingtoL+const.XDbindingtoMoRE-XDscompetingforbindingtoL
  as in:
  $$A\;=\;\alpha_{1}(P_{1wt3M}+\frac{P_{2wt2M}}{2}+\frac{P_{3wt1M}}{3}+\frac{P_{4wt0M}}{4})+\alpha_{2\;}P_{MoRE}-\alpha_{3}[{\left(\frac{P_{2wt2M}}{2}\right)}^{2}+{\left(\frac{P_{3wt1M}}{3}\right)}^{2}+{\left(\frac{P_{4wt0M}}{4}\right)}^{2}]$$
  Assumptions made: all-mutant P tetramers (P_0wt4M_) are bio-inactive (Figs 4E, 8A and 8C); all four P-XDs within a P tetramer function independently of each other; bioactivity requires XD binding to L and MoRE; both wild type and mutant XDs are equally MoRE binding competent (Fig 4B); the V463R mutation selectively impairs XD interaction with L only; and a single L binding-competent XD in the P tetramer is necessary and sufficient for bioactivity of the RdRP complex (Fig 8B-3 and 8D-1).

### Data fitting

Experimental measurements of relative RdRP bioactivities were fitted to the above models using Isqcurvefit, a nonlinear regression model in Optimization Toolbox of MATLAB R2017a (MathWorks). Goodness of fit was calculated using R^2^ values, defined as the proportion of the variance in the dependent variable that is predicable from the independent variables. Activity data sets for P (361–364)_Ala_ and P-V463R were assigned a default bioactivity data point A = 1 when ρ = 0.

### Statistical analyses

One or two-way ANOVA with Tukey’s post-hoc multiple comparisons tests or T-tests were used to assess statistical difference between samples, in all cases using the Prism 8 software package (Graph Pad). The nature of individual statistical tests applied is specified in each Figure legend, p value-intervals are shown. Wherever possible, graphical representations of experimental results show both the means of all biological replicates ± standard deviations (SDs) and individual biological repeats. All experiments were carried out in at least three biological repeats.

## Supporting information

S1 Fig Full-length L and truncated L_1708_ interact with P with equal efficiency. Cartoons provide a schematic overview of the L constructs. Numbers refer to amino acids. Immunoblots of input and co-precipitated material assessing P interaction with full-length and truncated L. Detection and quantitative analysis as in Fig 1C. Columns show means ± SD, symbols represent individual biological repeats (n = 3). Statistical analysis through unpaired T-test (NS, not significant).
(TIF)

S2 Fig Native PAGE analysis of P (361–364)_Ala_ and respectively denatured and native wild type P. SDS-PAGE shows immunoblots of the identical samples after denaturation and reduction.
(TIF)

S3 Fig RdRP minireplicon activity profile describing the effect of different relative amounts of wild type P-encoding plasmid DNA transfected. Amounts of plasmids encoding L and N were kept constant. Symbols show means of experimentally observed biological repeats ± SD (n = 3). Statistical analyses through one-way ANOVA with Dunnett’s multiple comparison test, relative to starting conditions (0.1 μg); (NS, not significant).
(TIF)

S4 Fig Minireplicon analysis of bioactivity of the C-terminally truncated P mutants depicted in Fig 3A. Columns represent mean relative RdRP activities, symbols show individual biological repeats ± SD (n = 3). Statistical analyses through one-way ANOVA and Tukey’s multiple comparison test (NS, not significant; ****, p ≤ 0.0001).
(TIF)

S5 Fig Effect of P (361–364)_Ala_ and P-V463R mutations on P binding to full-length L. Interaction analysis was carried out as specified in Fig 1C, using equally Flag epitope-tagged L_1708_ and full-length L as co-IP targets.
(TIF)

S6 Fig Multi-sequence alignments of P proteins of selected paramyxoviruses, representing genera of major clinical importance. Alignment with Clustal Omega algorithm (MeV P (NP_056919.1); CDV P (AIN44014.1); RV P (AAB23268.1); hendra virus (HeV) P (APT69525.1); nipah virus (P) P (QBQ56717.1); SeV P (AAB06279.1); HPIV-1 P (AAL89402.1); HPIV-3 P (BAA00921.1); mumps virus (MuV) P (BAA00260.1), HPIV-2 P (ART66806.1), and PIV-5 (YP_138512.1). α-helical regions are highlighted above the sequences and heptad repeats in P-OD indicated; numbering refers to MeV. P residues 361–364 are highlighted in green, color-coding of individual residues in P-XD according to the scheme used in Fig 4A.
(TIF)

S7 Fig Homo- and hetero-typic interaction of MeV L with P derived from MeV or CDV, respectively. Interaction analysis, immuno-detection and signal quantitation in Fig 1C. Columns show means ± SD, symbols represent individual biological repeats (n = 3). Statistical analysis through unpaired T-test (NS, not significant).
(TIF)

S8 Fig Minireplicon analysis of relative bioactivity of P mutants with alanine substitutions in conserved patches in the connector domain (specified in Fig 7A; red numbers). Columns show means ± SD, symbols represent individual biological repeats (n = 3). Statistical analysis through one-way ANOVA and Tukey’s multiple comparison test (NS, not significant; ***, p ≤ 0.001; ****, p ≤ 0.0001).
(TIF)
