# Supplementary figures and images for: Bipartite interface of the measles virus phosphoprotein X domain with the large polymerase protein regulates viral polymerase dynamics
Source: PLoS Pathog. 2019 Aug 5;15(8):e1007995. doi: 10.1371/journal.ppat.1007995 (PMC6695210; doi:10.1371/journal.ppat.1007995)

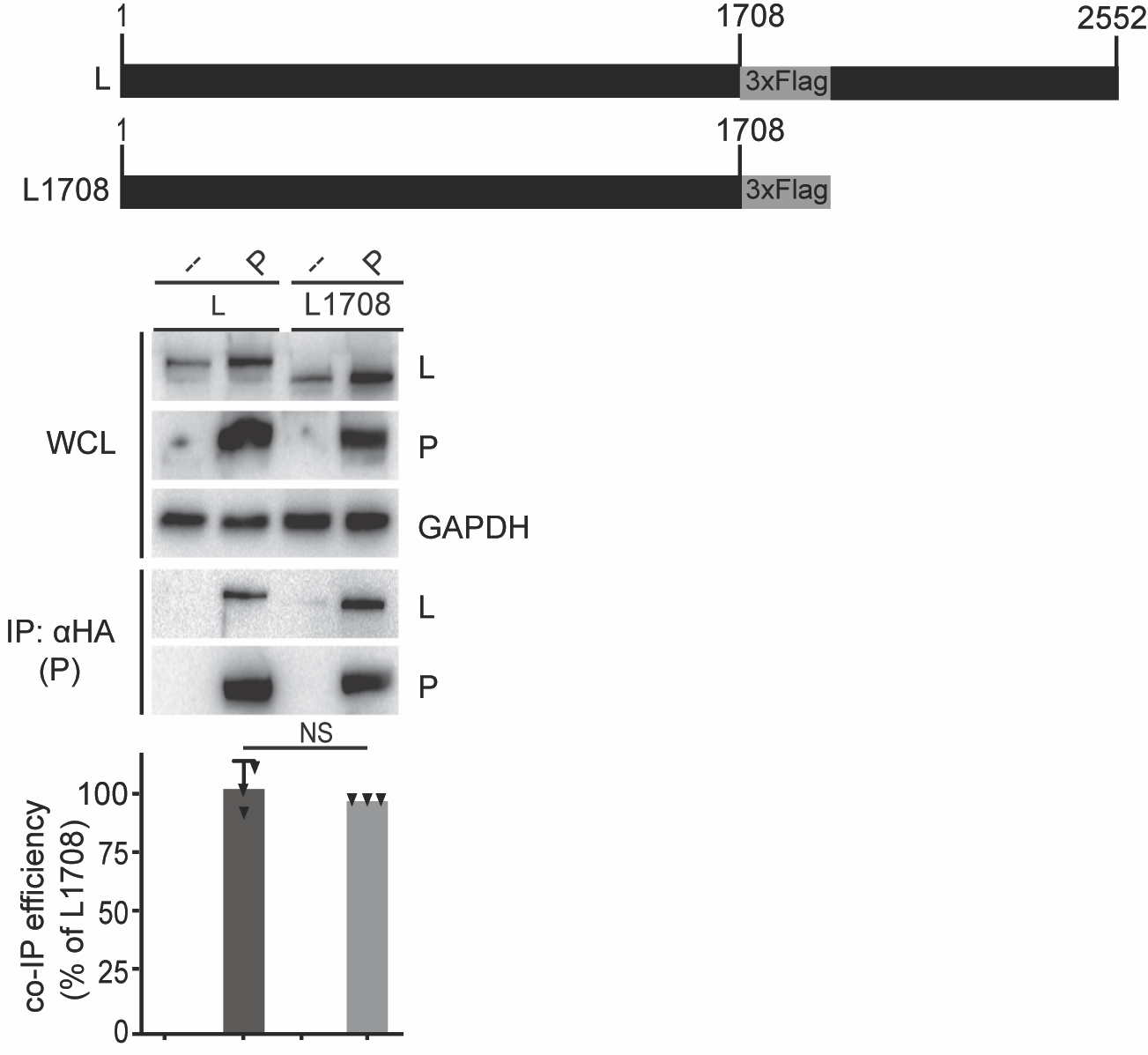

Supplement: S1 Fig — Cartoons provide a schematic overview of the L constructs. Numbers refer to amino acids. Immunoblots of input and co-precipitated material assessing P interaction with full-length and truncated L. Detection and quantitative analysis as in Fig 1C. Columns show means ± SD, symbols represent individual biological repeats (n = 3). Statistical analysis through unpaired T-test (NS, not significant). (TIF) [file ppat.1007995.s001.tif]

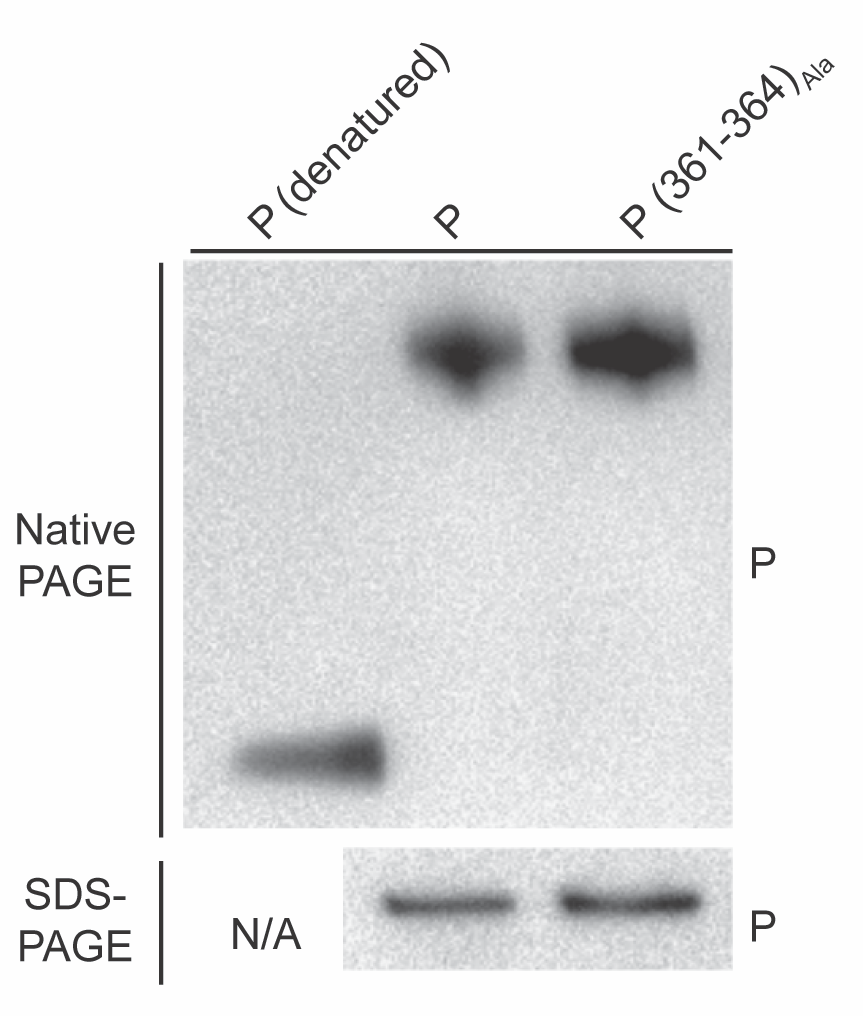

Supplement: S2 Fig — SDS-PAGE shows immunoblots of the identical samples after denaturation and reduction. (TIF) [file ppat.1007995.s002.tif]

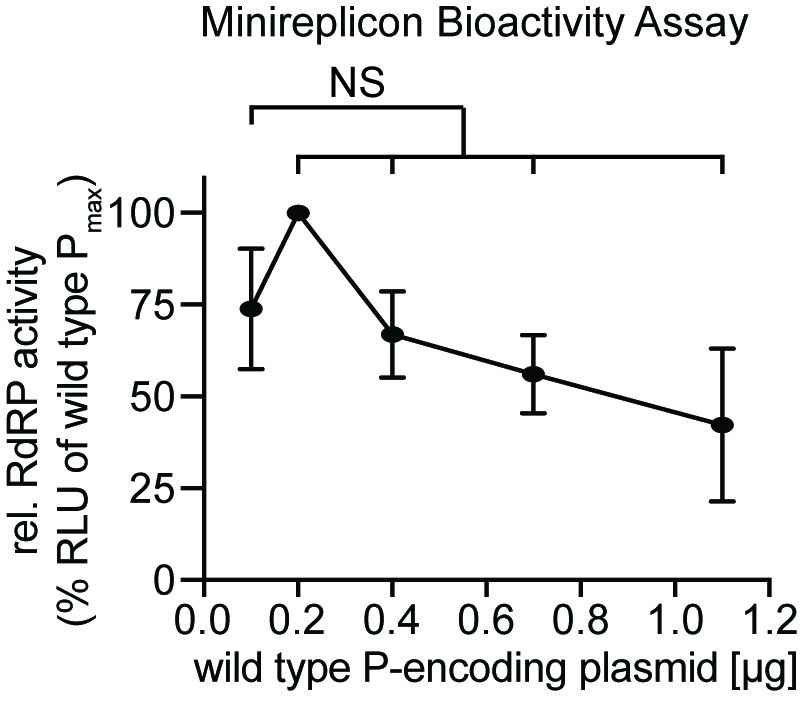

Supplement: S3 Fig — Amounts of plasmids encoding L and N were kept constant. Symbols show means of experimentally observed biological repeats ± SD (n = 3). Statistical analyses through one-way ANOVA with Dunnett’s multiple comparison test, relative to starting conditions (0.1 μg); (NS, not significant). (TIF) [file ppat.1007995.s003.tif]

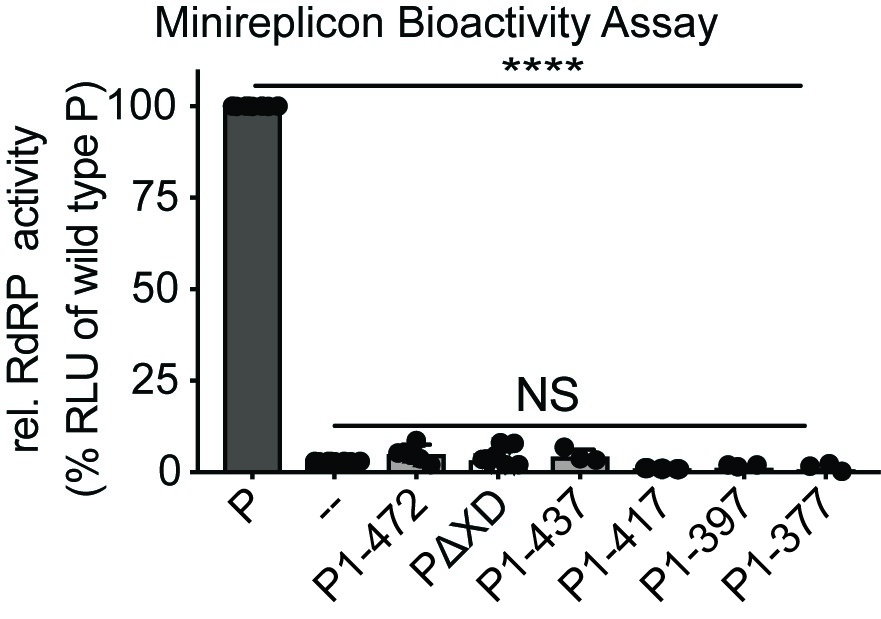

Supplement: S4 Fig — Columns represent mean relative RdRP activities, symbols show individual biological repeats ± SD (n = 3). Statistical analyses through one-way ANOVA and Tukey’s multiple comparison test (NS, not significant; ****, p ≤ 0.0001). (TIF) [file ppat.1007995.s004.tif]

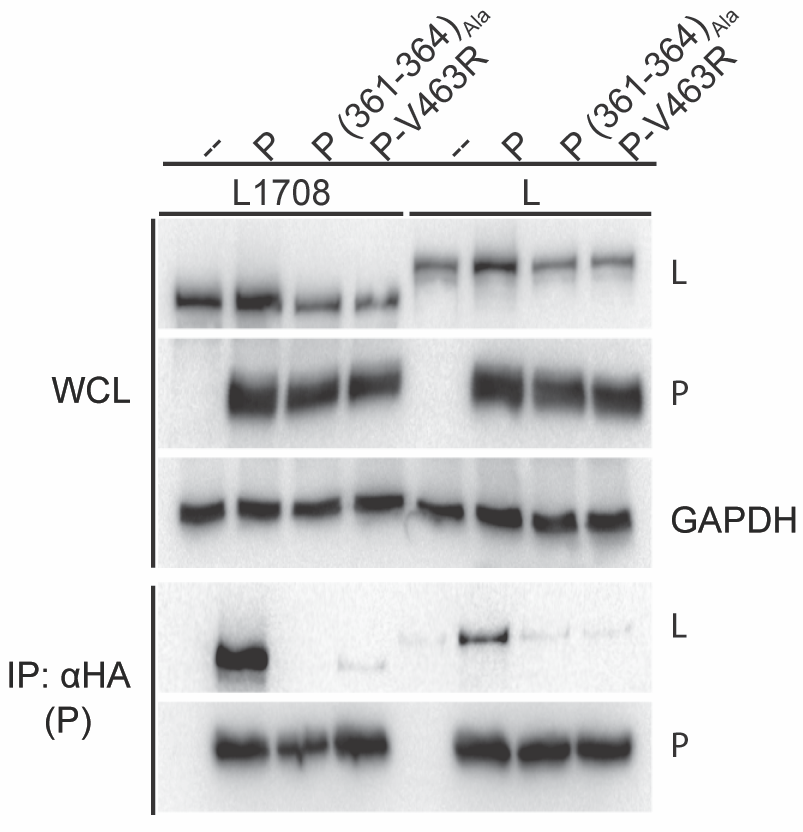

Supplement: S5 Fig — Interaction analysis was carried out as specified in Fig 1C, using equally Flag epitope-tagged L1708 and full-length L as co-IP targets. (TIF) [file ppat.1007995.s005.tif]

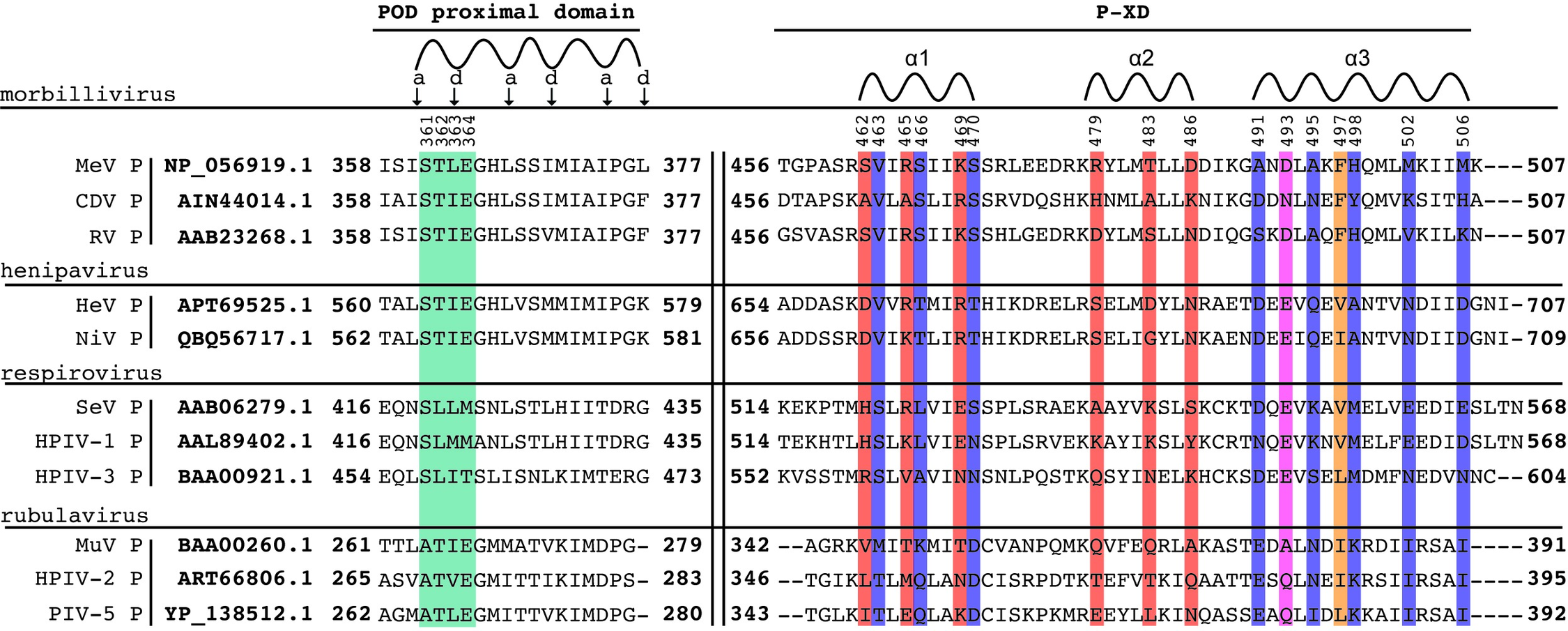

Supplement: S6 Fig — Alignment with Clustal Omega algorithm (MeV P (NP_056919.1); CDV P (AIN44014.1); RV P (AAB23268.1); hendra virus (HeV) P (APT69525.1); nipah virus (P) P (QBQ56717.1); SeV P (AAB06279.1); HPIV-1 P (AAL89402.1); HPIV-3 P (BAA00921.1); mumps virus (MuV) P (BAA00260.1), HPIV-2 P (ART66806.1), and PIV-5 (YP_138512.1). α-helical regions are highlighted above the sequences and heptad repeats in P-OD indicated; numbering refers to MeV. P residues 361–364 are highlighted in green, color-coding of individual residues in P-XD according to the scheme used in Fig 4A. (TIF) [file ppat.1007995.s006.tif]

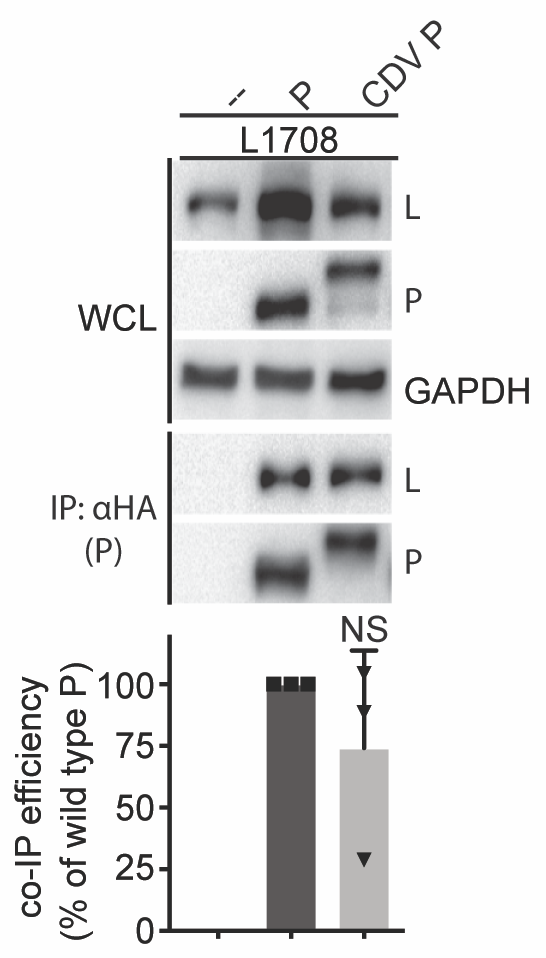

Supplement: S7 Fig — Interaction analysis, immuno-detection and signal quantitation in Fig 1C. Columns show means ± SD, symbols represent individual biological repeats (n = 3). Statistical analysis through unpaired T-test (NS, not significant). (TIF) [file ppat.1007995.s007.tif]

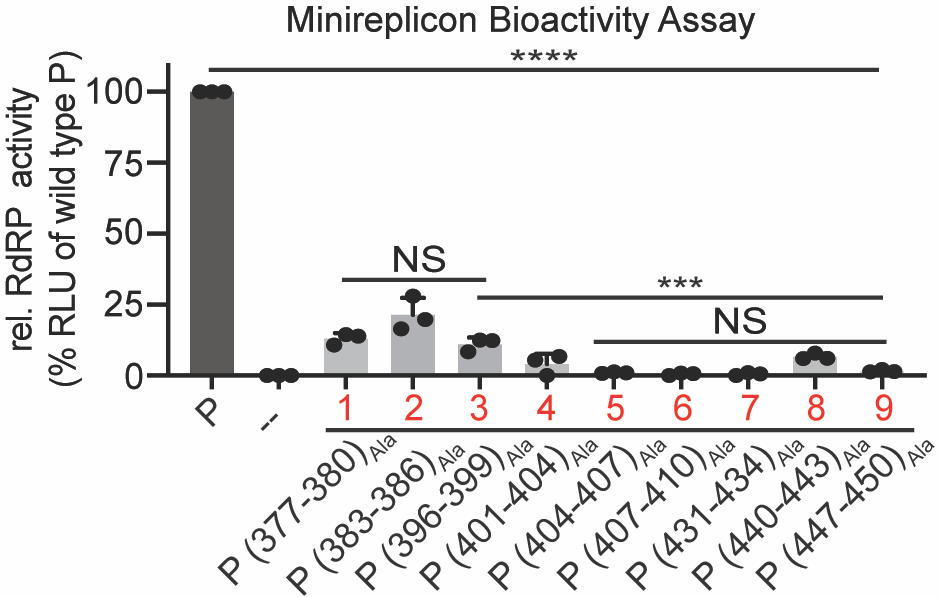

Supplement: S8 Fig — Columns show means ± SD, symbols represent individual biological repeats (n = 3). Statistical analysis through one-way ANOVA and Tukey’s multiple comparison test (NS, not significant; ***, p ≤ 0.001; ****, p ≤ 0.0001). (TIF) [file ppat.1007995.s008.tif]
